# Novel Lysosome-Targeting Fluorescence Off-On Photosensitizer for Near-Infrared Hypoxia Imaging and Photodynamic Therapy In Vitro and In Vivo

**DOI:** 10.3390/molecules27113457

**Published:** 2022-05-27

**Authors:** Shangli Ding, Mingyan Yang, Jiajia Lv, Hongyu Li, Gang Wei, Jie Gao, Zeli Yuan

**Affiliations:** 1 Key Laboratory of Basic Pharmacology of Ministry of Education and Joint International Research Laboratory of Ethnomedicine of Ministry of Education, Zunyi Medical University, No.6 West Xuefu Road, Xinpu District, Zunyi City 563000, China; m18608538251@163.com (S.D.); myyoung@whut.edu.cn (M.Y.); jiajialv@zmu.edu.cn (J.L.); lihongyu@iccas.ac.cn (H.L.); 2 Key Laboratory of Biocatalysis & Chiral Drug Synthesis of Guizhou Province, School of Pharmacy, Zunyi Medical University, No.6 West Xuefu Road, Xinpu District, Zunyi City 563000, China; 3 Guizhou International Scientific and Technological Cooperation Base for Medical Photo-Theranostics Technology and Innovative Drug Development, Zunyi Medical University, No.6 West Xuefu Road, Xinpu District, Zunyi City 563000, China; 4 CSIRO Mineral Resources, P.O. Box 218, Lindfield, NSW 2070, Australia

**Keywords:** photodynamic therapy, hypoxia, nitroreductase, lysosome, near-infrared, cyanine

## Abstract

Photodynamic therapy (PDT) has emerged as a new antitumor modality. Hypoxia, a vital characteristic of solid tumors, can be explored to stimulate the fluorescence response of photosensitizers (PSs). Considering the characteristics of PDT, the targeting of organelles employing PS would enhance antitumor effects. A new multifunctional cyanine-based PS (CLN) comprising morpholine and nitrobenzene groups was prepared and characterized. It generated fluorescence in the near-infrared (NIR) region in the presence of sodium dithionite (Na_2_S_2_O_4_) and nitroreductase (NTR). The response mechanism of CLN was well investigated, thus revealing that its obtained reduction product was CLNH. The obtained fluorescence and singlet oxygen quantum yield of CLNH were 8.65% and 1.60%, respectively. Additionally, the selective experiment for substrates indicated that CLN exhibited a selective response to NTR. Thus, CLN fluorescence could be selectively switched on and its fluorescence intensity increased, following a prolonged stay in hypoxic cells. Furthermore, fluorescence colocalization demonstrated that CLN could effectively target lysosomes. CLN could generate reactive oxygen species and kill tumor cells (IC50 for 4T1 cells was 7.4 μM under a hypoxic condition), following its response to NTR. NIR imaging and targeted PDT were finally applied in vivo.

## 1. Introduction

Many strategies, particularly photodynamic therapy (PDT), are emerging as new and noninvasive therapy modalities for cancer treatment [1,2,3]. PDT relies on specific light-generated reactive oxygen species (ROS) in the presence of photosensitizers (PSs) to kill cancer cells. Additionally, PSs generally possess certain fluorescence-emitting abilities and can facilitate antitumor effects and image-guided therapy during PDT [4,5]. However, the fluorescence of PS is “always-on”, resulting in low signal-to-noise ratios and poor imaging qualities [6,7,8]. Moreover, PS-generated ROS exhibit a short life (0.03–0.18 ms) and limited distance of action (0.01–0.02 μm) [9,10,11]. Therefore, the location of the PS-generated ROS is directly proportional to the anticancer effect. If vital or fragile organelles can be precisely enriched with PSs, the concentrated ROS will focus on these “lethal sites” of cancer cells and be more lethal than randomly dispersed PSs in tumor tissue or cells [11,12,13,14,15]. Organelles are integral to maintaining cellular structures and functions, and their damage will result in cellular dysfunction, apoptosis, necrosis, etc. Therefore, the fluorescence of an ideal PS must be able to “turn on” selectively under certain conditions and target organelles to enhance tumor ablation.

A series of physiological stimuli, such as hypoxia, acidity, enzymes, redox substances, and adenosine triphosphate in tumor microenvironments, have been explored to develop fluorophores and PSs with fluorescence “turn on” properties [16,17,18,19,20,21,22]. Among them, hypoxia, i.e., low oxygen levels (0.02–2% O_2_) in tissues, is considered a vital characteristic of solid tumors [23]. Particularly, nitroreductase (NTR), a flavin protease, is overexpressed in hypoxic cells. NTR can readily reduce nitroaromatics into the corresponding amines under conditions in which nicotinamide adenine dinucleotide/nicotinamide adenine dinucleotide phosphate (NADH/NADPH) act as the hydrogen donor [24]. Therefore, an upregulated level of NTR is considered a tumor biomarker [25]. Previous studies demonstrated that nitroaromatics are excellent substrates for assessing NTR levels, which have been widely employed to develop noninvasive probes for hypoxic tumor imaging [25,26], as well as PSs for antitumor efficiency [27,28].

Lysosomes, one of the most essential and common organelles in cells, account for the degradation and recycling of different macromolecules; they are known as “garbage collection stations” [29,30,31]. Further, lysosomes play an essential role in cell signaling and migration, as well as in activating apoptosis and necrosis [31,32]. Once disrupted, lysosomes release protons and different hydrolases, thereby inducing apoptosis and eventual cell death [33,34]. Compared with normal cells, cancer cells contain more lysosomes [35] that can be employed as targets for PSs with high therapeutic indices. Based on the crucial physiological functions of lysosomes, as well as their quantitative advantages in tumor cells, a series of lysosome-targeting PSs have been designed to enhance antitumor efficiency [36,37,38,39,40,41,42,43].

**Scheme 1:**
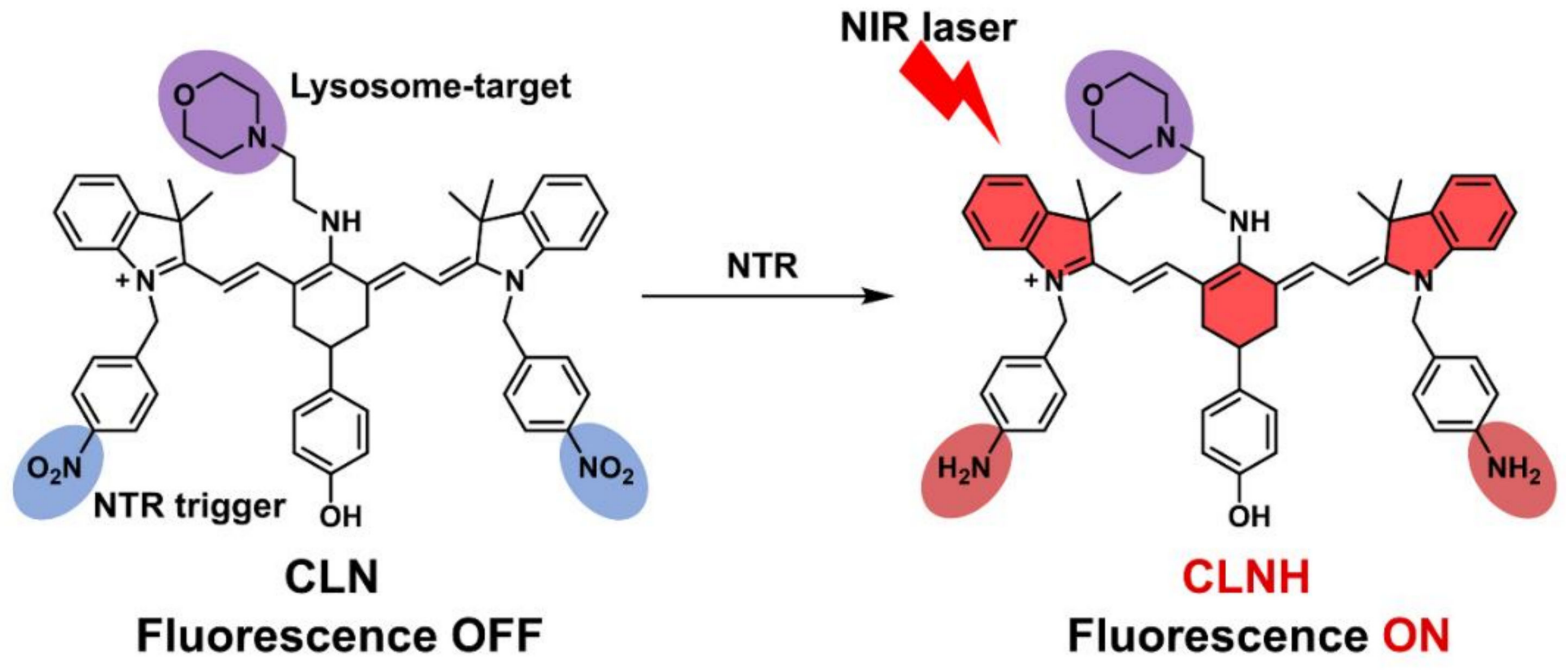
Targeting and NIR-florescence-activation mechanisms of the CLN. The cyanine fluorophore is marked in bright red in the structure of CLNH; the lysosome-targeted morpholine group is labeled in purple; the nitrobenzene groups as an NTR-response unit are marked in blue in the structure of CLN, which are reduced to amino groups after being triggered by NTR, marked in dark red in the structure of CLNH.

Here, we designed and prepared a novel multifunctional cyanine-based derivative (CLN) as PS for near-infrared (NIR) image-guided antitumor PDT (Scheme 1). CLN comprised three parts: (1) the cyanine fluorophore that was selected as the PS core to achieve NIR PDT, (2) a morpholine group that was modified on meso-cyanine to target lysosomes, and (3) two nitrobenzene groups that were attached to each side of cyanine to serve as an NTR-response unit to turn on the fluorescence. CLN absorbs light in the NIR region but with almost no fluorescence, owing to the photoinduced electron transfer (PET) effect of the nitrobenzene moiety. Further, NTR can selectively and sensitively turn on CLN fluorescence. Cellular experiments demonstrated the effectiveness of CLN in targeting lysosomes. Furthermore, tumor hypoxia imaging by CLN was performed on cellular and animal hypoxia models. CLN had been successfully applied to anticancer PDT in vitro and in vivo. This study demonstrated the suitability of CLN as an ideal multifunctional PS.

## 2. Results and Discussions

### 2.1. Design and Synthesis of the Photosensitizer CLN

**Scheme 2:**
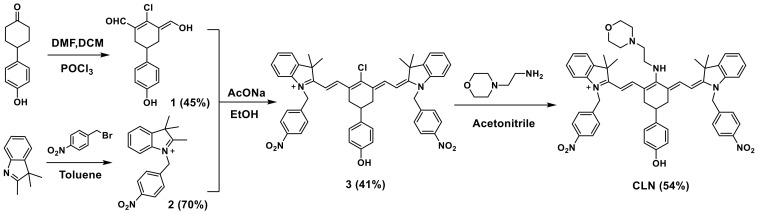
Synthetic route of the CLN.

Three factors were considered when designing lysosome-targeted NTR-stimulated NIR PS with fluorescence responses. First, the absorption and fluorescence emission wavelengths of the fluorescent unit of PS were preferably in the NIR region to increase the light-penetration depth, reduce the background, and improve the imaging quality and PDT effect [44]. Second, morpholine, which is known to facilitate the localization of PS in the cellular lysosome, as well as enhance the cell-killing effect of PS, was selected as the lysosome-targeting group [11]. Third, nitrobenzene, which has exhibited high sensitivity to NTR in vitro and in vivo, was selected as the recognition site for NTR. Additionally, the fluorescence of PS was quenched via the PET effect and restored after responding to NTR [16]. Here, we prepared our novel cyanine-based lysosomal PS (CLN) by introducing the morpholine and nitrobenzene groups, which could selectively locate lysosomes, demonstrate a specific response to NTR, and facilitate NIR fluorescence imaging and PDT (Scheme 1). CLN was obtained by a four-step synthesis (Scheme 2). Further, the structures of the intermediates and CLN were characterized by nuclear magnetic resonance (NMR) spectroscopy and high-resolution mass spectrometry (HRMS) (Figures S1–S8).

### 2.2. Photophysical Properties

**Figure 1:**
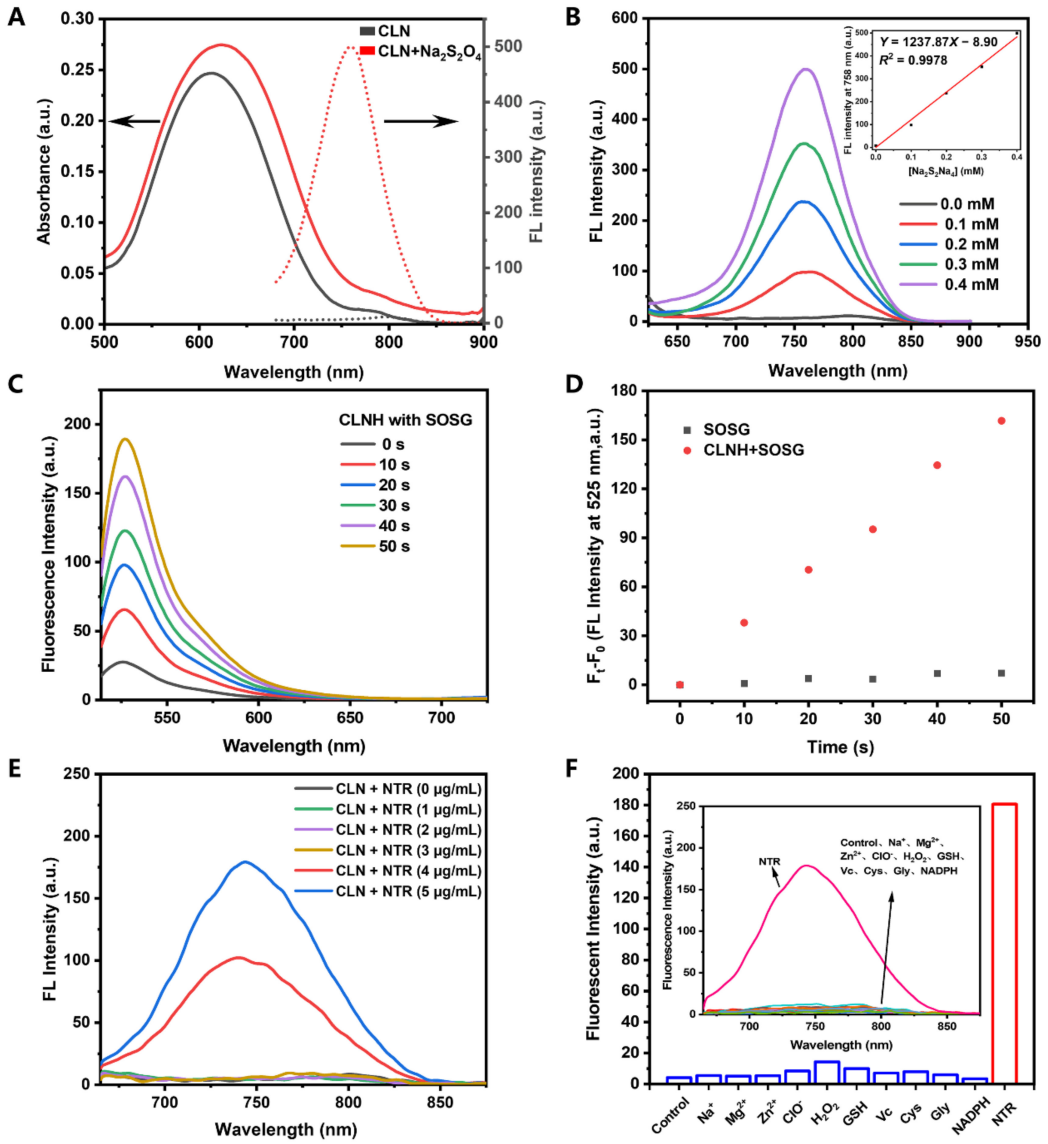
(**A**) Absorption (solid lines) and fluorescence spectra (dotted lines) of CLN (10 μM) without (black) or with (red) Na_2_S_2_O_4_ (0.4 mM). (**B**) Fluorescence spectra of CLN (10 μM) in different concentrations of Na_2_S_2_O_4_ (0–0.4 mM). Inset: The linear correlation of the fluorescence intensities at 758 nm with the concentrations of Na_2_S_2_O_4_. (**C**) Fluorescence spectra of SOSG (2 μM) in the CLN solution (10 μM) after NIR laser irradiation (660 nm, 0.2 W/cm^2^) at different time points (0, 10, 20, 30, 40, and 50 s). (**D**) Changes in the fluorescence intensities at 525 nm vs. the irradiation time. (**E**) Changes in the fluorescence spectra of CLN (10 μM) response to different NTR concentrations (0–5 µg/mL) with NADPH (500 μM) in PBS (10 mM, pH 7.4) containing 10% EtOH at 37 °C after 30 min of incubation. (**F**) Selectivities of the fluorescence responses of CLN (10 μM) to different species: NaCl (10 mM), MgCl_2_ (10 mM), ZnSO_4_ (10 mM), ClO^−^ (10 mM), H_2_O_2_ (10 mM), GSH (1 mM), Vc (1 mM), Cys (1 mM), Gly (1 mM), NADPH (500 μM), and NTR (5 μg/mL in the presence of NADPH (500 μM)) in PBS buffer at 37 °C in the presence of CLN (10 μM). Control: CLN (10 μM). All the samples were tested after 30 min of incubation.

Figure 1A shows that the maximum absorption wavelength of CLN was 611 nm. Expectedly, almost no fluorescence was detected before the CLN response. The absorption and fluorescence spectra of CLN were recorded under different pH conditions (pH 2–12), as shown in Figure S11A,B. Notably, the fluorescence signals of CLN were extremely weak, due to the PET effect of the nitrobenzene moiety. Further, the pKa value of 6.65 for “-NH-” at meso-CLN was obtained via fitting the absorbance at 411 nm vs. values of pH (Figure S11C). Thus, we first utilized sodium dithionite (Na_2_S_2_O_4_) to simulate a reducing environment since it is a reliable reducing agent for aromatic nitro groups. After adding Na_2_S_2_O_4_, the maximum absorption wavelength of CLN shifted from 611 to 623 nm, indicating the probable production of a new product. Concurrently, an intense fluorescence signal was generated in the NIR region (the fluorescence intensity at the maximum wavelength (758 nm) was 71 times higher than before). Moreover, the fluorescence quantum yields of CLN were 0.13% and 8.65% before and after the addition of Na_2_S_2_O_4_ in a phosphate-buffered saline (PBS) solution, respectively. Finally, the mechanism of the generation of fluorescence was confirmed via NMR titration and MS. Following the addition of Na_2_S_2_O_4_ into the CLN solution (DMSO-*d*_6_), a new peak, which could be a signal for the hydrogen of aromatic amines, appeared at 5.00 ppm (Figure S9). Furthermore, the mass peak, reflecting the reduction product (CLNH), was generated at *m*/*z* = 852.5080 (Figure S10). Thus, the above results indicated that the fluorescence was generated by the reduction of the CLN-attached nitrobenzene groups into aminobenzene groups in the presence of Na_2_S_2_O_4_, which also generated CLNH. Scheme 1 outlines the NIR-florescence-activation mechanism of CLN. Further, CLN for detecting Na_2_S_2_O_4_ was investigated. Figure 1B shows that the concentration of Na_2_S_2_O_4_ and the fluorescence intensity at 758 nm exhibited good linear fitting (0–0.4 mM), demonstrating the quantitative feasibility of Na_2_S_2_O_4_. Following the above quantification curve, Na_2_S_2_O_4_ exhibited a detection limit of 0.13 μM.

The singlet oxygen probe, Singlet Oxygen Sensor Green (SOSG), was employed to evaluate the singlet oxygen generation of CLNH (the reduction product CLN). Figure 1C shows that the fluorescence signals of SOSG increased with the laser irradiation time in the CLNH solution. Under the same condition, SOSG displayed almost no fluorescence under laser irradiation (Figure 1D). Employing methylene blue as the standard (0.39 in water), the obtained singlet oxygen quantum yield of CLNH was 1.60% [45]. All the optical data of CLN and CLNH in PBS were shown in Table S1, including the maximum absorption and emission wavelength, Stokes shifts, molar extinction coefficient, and quantum yields of fluorescence and singlet oxygen.

Inspired by the above results, we compared the changes in the fluorescence of CLN before and after its response to NTR (Figure 1D). CLN barely displayed fluorescence without the addition of NTR. However, the fluorescence intensities at 744 nm demonstrated ~17- and 29-fold increases at 4 and 5 μg/mL concentrations of NTR, respectively. Moreover, the maximum emission (744 nm) of CLN reduction by NTR exhibited blue shift compared to the CLN reduction by Na_2_S_2_O_4_ (758 nm), which was due to the different solvent environments, one in PBS/10% EtOH and the other in PBS buffer (Figure S11D). To investigate the selectivity of CLN for NTR, the fluorescence spectra and intensities at 744 nm were recorded in the presence of biological species, including glutathione (GSH), vitamin C (Vc), cysteine (Cys), glycine (Gly), and nicotinamide adenine dinucleotide phosphate (NADPH), as well as other species, including inorganic salts (NaCl, MgCl_2_, and ZnSO_4_) and reactive oxygen/nitrogen species (ClO^−^, and H_2_O_2_). Figure 1D shows that only the “NTR” group exhibited a strong fluorescence signal at 744 nm. No significant fluorescence was generated in the other groups, confirming that CLN exhibited a highly selective response to NTR in complicated environments.

### 2.3. Fluorescence Imaging in Hypoxic Cells

**Figure 2:**
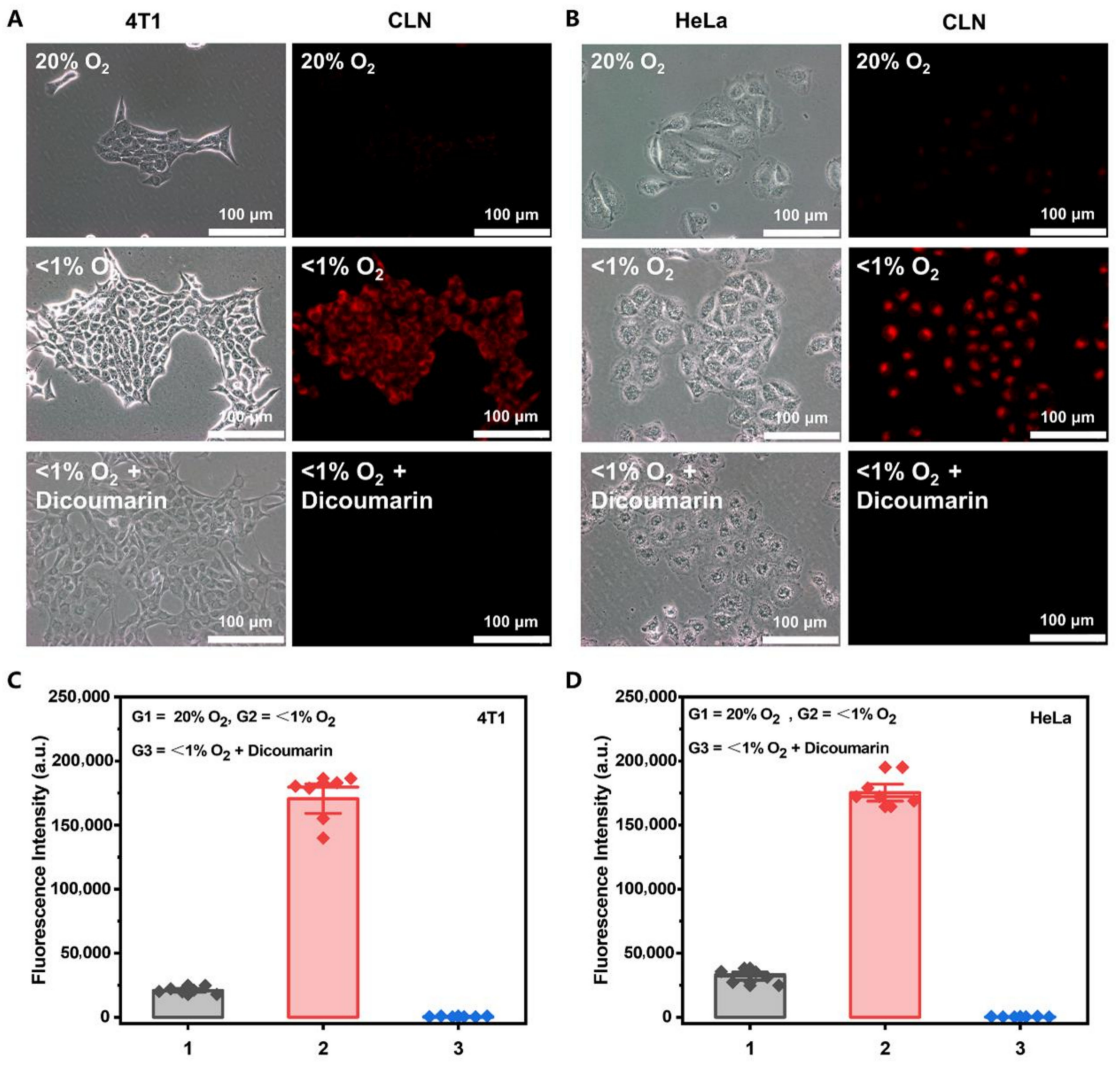
(**A**,**B**) Fluorescence imaging and (**C**,**D**) relative cell fluorescence intensities of CLN (5 μM) in the 4T1 and HeLa cells under normoxic (20% O_2_) and hypoxic (<1% O_2_) conditions without or with the NTR inhibitor (dicoumarin (0.5 mM)), respectively. These cells were incubated for 3 h with 5 µM CLN, after which they were kept under normoxic (20% O_2_) and hypoxic conditions (<1% O_2_) for 8 h, followed by the incubation of the hypoxic group with 0.5 mM dicoumarin for 1 h before hypoxia. The fluorescence imaging of these cells was performed with an Olympus IX73 (scale bar = 100 μm).

Considering the excellent performance of our CLN, we evaluated its imaging performance under cellular hypoxic conditions. We investigated the selectivity of CLN imaging by more precisely controlling the oxygen content and adding NTR inhibitors (dicoumarin) [46]. In this test, the 4T1 and HeLa cells were first incubated with CLN for 3 h. Thereafter, the cells were incubated under normoxic (20% O_2_) and hypoxic conditions (<1% O_2_) for 8 h. Additionally, a hypoxic group was incubated with 0.5 mM dicoumarin for 1 h before hypoxia. Figure 2A,B show that almost no fluorescent signal was observed in the normoxic cells. Expectedly, the bright red fluorescence signal appeared in the hypoxia ones. Under the hypoxia conditions, the red fluorescence disappeared when dicoumarin was added to the cells. The statistics of the fluorescence intensities (Figure 2C,D) corroborated the cell imaging results. These results indicated that the fluorescence intensity of CLN was closely related to the degree of oxygen concentration and could selectively respond to NTR.

### 2.4. Subcellular Localization of CLN

**Figure 3:**
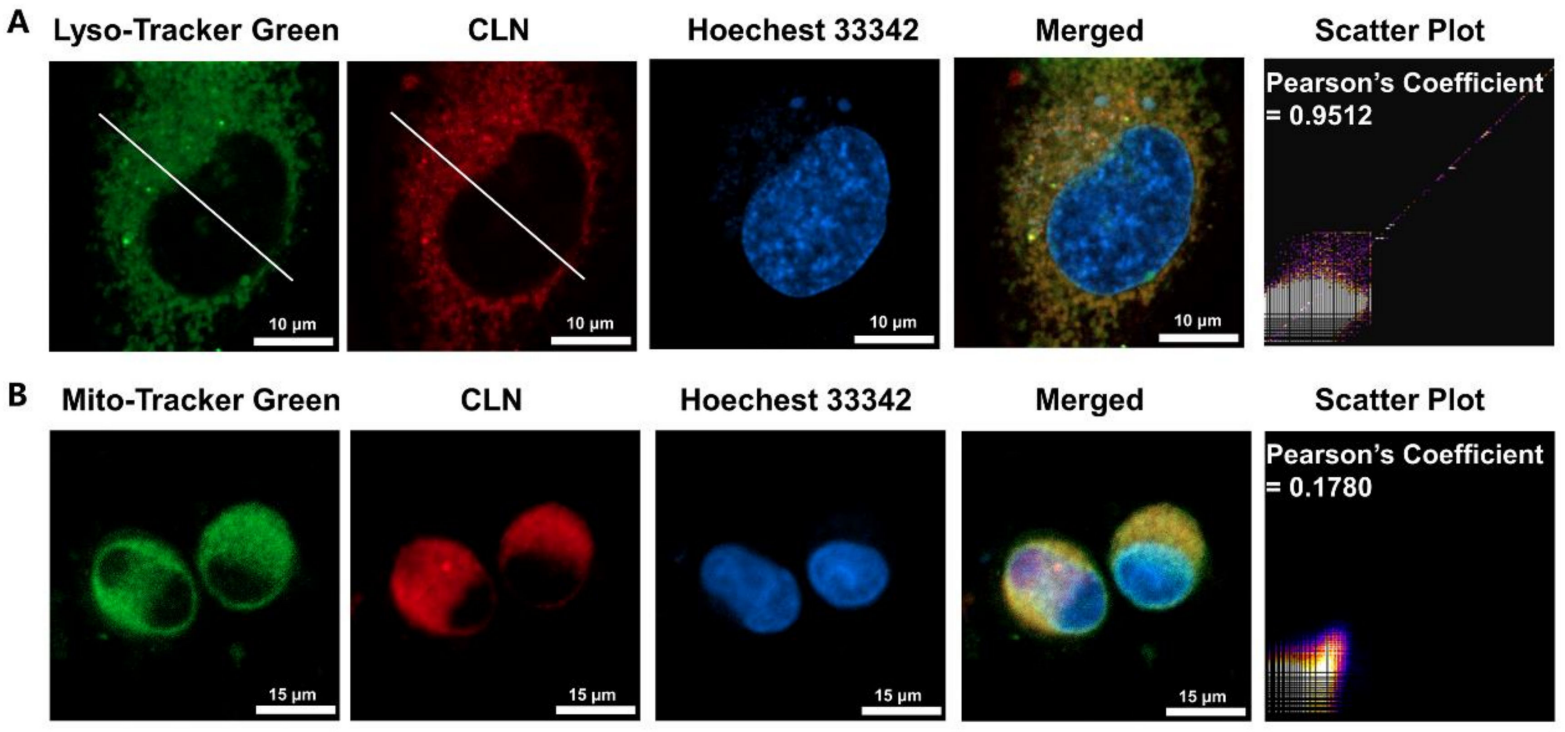
Subcellular colocalization assays of CLN (5 μM) in the HeLa cells under hypoxic conditions. The green channel (Mito-Tracker Green) was excited at 488 nm, the blue channel (Hoechst 33342) was excited at 405 nm, and the red channel (CLN) was excited at 640 nm ((**A**) Scale bar = 10 μm; (**B**) Scale bar = 15 μm).

To confirm that CLN could target lysosomes upon entering the cells, we selected Lyso-Tracker Green, a commercial lysosomal probe, for the colocalization imaging experiments. Further, Mito-Tracker Green, a mitochondria-targeting probe, was employed for the colocalization studies. Figure 3A shows that the fluorescence signal of CLN overlapped well with that of Lyso-Tracker Green in the HeLa cells under hypoxic conditions (the Pearson coefficient was 0.95). However, the region of the distribution of the CLN fluorescence was significantly different from that of Mito-Tracker Green (the Pearson correlation coefficient was 0.18). Thus, we confirmed that CLN could target lysosomes in cells.

### 2.5. In Vitro Generation of ROS and the Phototoxicity of CLN

**Figure 4:**
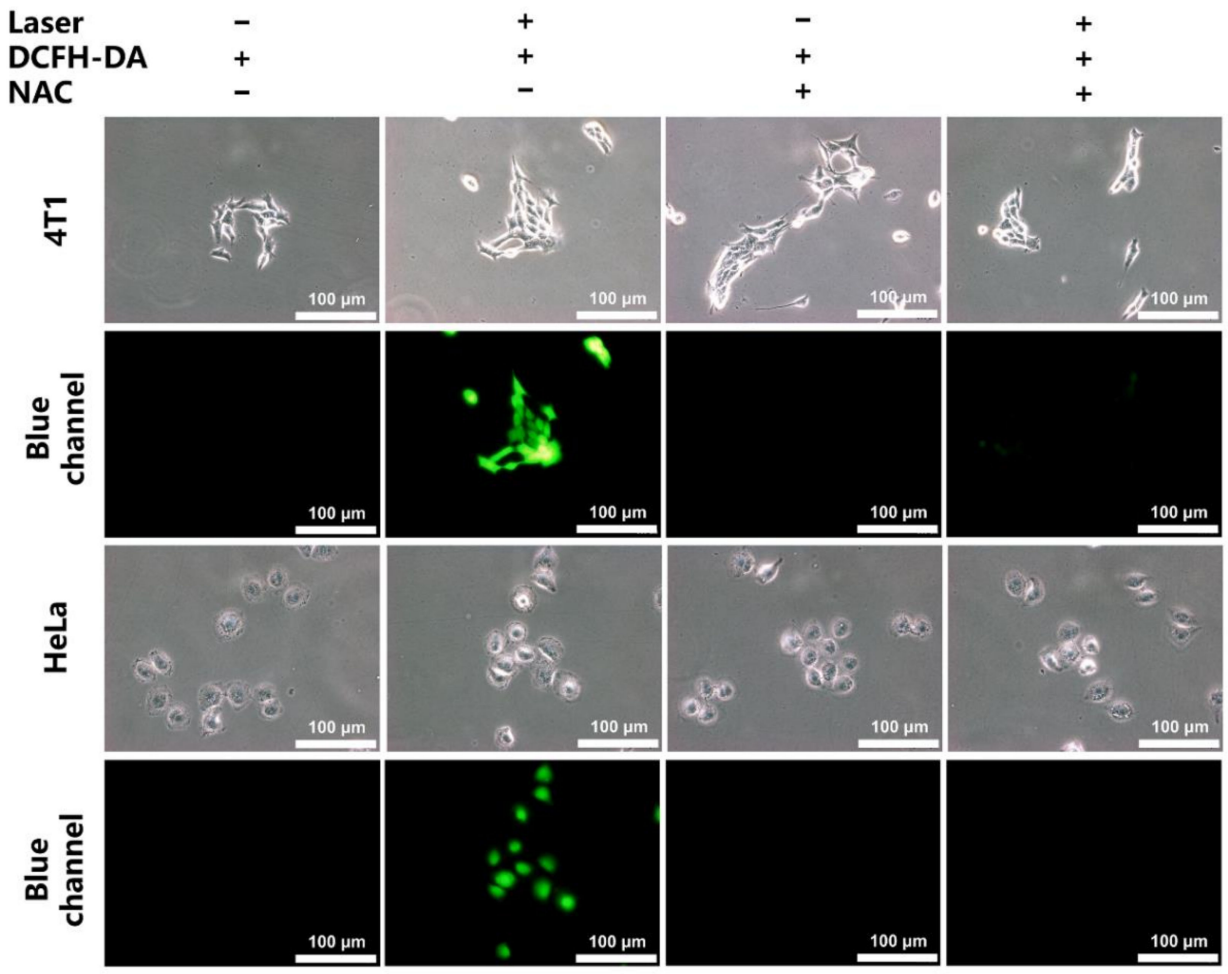
Cellular ROS levels of CLN (5 μM) in the 4T1 and HeLa cells employing a 5-μM 2′,7′-dichlorofluorescein diacetate (DCFH-DA) probe. The laser used a 660 nm (200 mW/cm^2^) laser light and 5 mM *N*-acetyl-L-cysteine (NAC) as reactive oxygen species scavenger (scale bar = 100 μm).

The intracellular generation of ROS in the 4T1 and HeLa cells was further investigated employing DCFH-DA as the intracellular ROS probe. After the laser irradiation without any other treatment, the CLN-treated cells displayed significant green fluorescence signals, indicating that CLN generated ROS in the cells (Figure 4). After adding N-acetyl-L-cysteine, the ROS scavenger, the green fluorescent signal disappeared.

After treating the 4T1 cells with CLN for 24 h in a normoxic environment, they were irradiated with a light-emitting diode (LED) light (220.7 mW/cm^2^) for 10 min to investigate its phototoxicity. The cell viability of the tested tumor cells decreased continuously with the increasing CLN concentrations, exhibiting significant cytotoxicity (Figure S12A). The IC50 value of CLN for 4T1 was 0.7 μM, indicating that CLN exhibited phototoxicity and potential application for PDT. More crucially, CLN still exerted an excellent killing effect on the 4T1 cells under hypoxic conditions (IC50 = 7.4 μM, Figure S12B). Furthermore, CLN did not exhibit significant dark toxicities in the concentration ranges of 0–1.6 and 0–12 µM under normoxic and hypoxia conditions, respectively (Figure S12).

### 2.6. In Vivo Imaging and Photo-Theranostic Study of CLN

**Figure 5:**
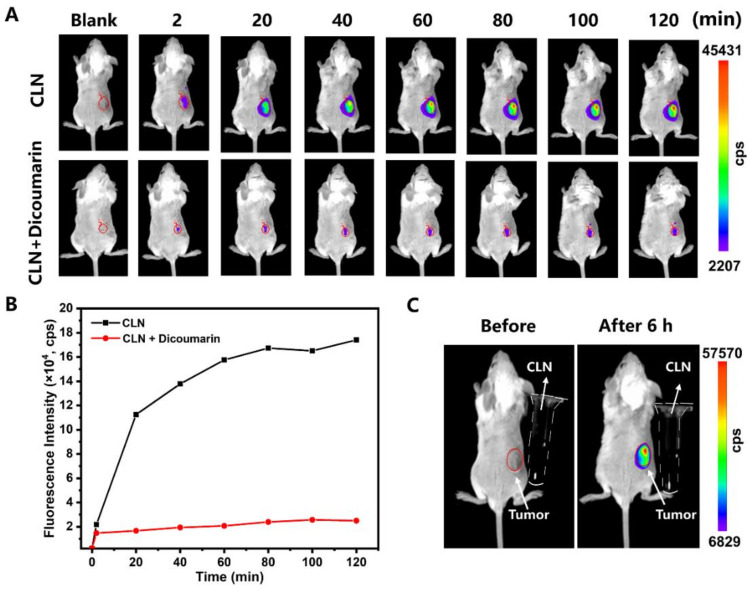
(**A**) Fluorescence imaging of the 4T1 tumor-bearing BALB/c mice at 2, 20, 40, 60, 80, 100, and 120 min after the intratumor injections of CLN (2.5 μmol/kg) and CLN (2.5 μmol/kg) + dicoumarin (0.5 mM). (**B**) Fluorescence intensities of the “CLN” and “CLN + Dicoumarin” groups at the tumor site vs. time (*n* = 3). (**C**) Fluorescence imaging of the 4T1 tumor-bearing BALB/c mice before and after 6 h of CLN (2.5 μmol/kg) injection and CLN solution in the Eppendorf tubes (the dotted outline; 500 μM, Tris buffer, 10 mM, pH = 7.4, containing 10% DMSO).

Motivated by the above results, we evaluated whether CLN could respond to NTR in vivo. The BALB/c mice bearing 4T1 tumors were established and randomly divided into two groups comprising three mice each. The groups were named “CLN” and “CLN + Dicoumarin”. CLN was directly injected into the tumors of the mice in the “CLN” group, after which they were imaged at different times (2, 20, 40, 60, 80, 100, and 120 min) by an in vivo imaging system. Regarding the “CLN + Dicoumarin” group, the same procedure as that of the “CLN” group was performed after injecting dicoumarin into their tumors for 10 min. Figure 5A,B show that the fluorescence signals of the tumors of the “CLN” group increased gradually and reached their maximum at 80 min. Expectedly, the fluorescence signals of the tumor target in the “CLN + Dicoumarin” group that was treated with the NTR inhibitor were extremely weaker. The “CLN” group exhibited strong fluorescence signals after 6 h of the intratumor injection of CLN (Figure 5C). The CLN solutions did not exhibit any fluorescence signal under the same conditions, thus confirming that CLN could respond to NTR, which selectively turned on its fluorescence in vivo.

**Figure 6:**
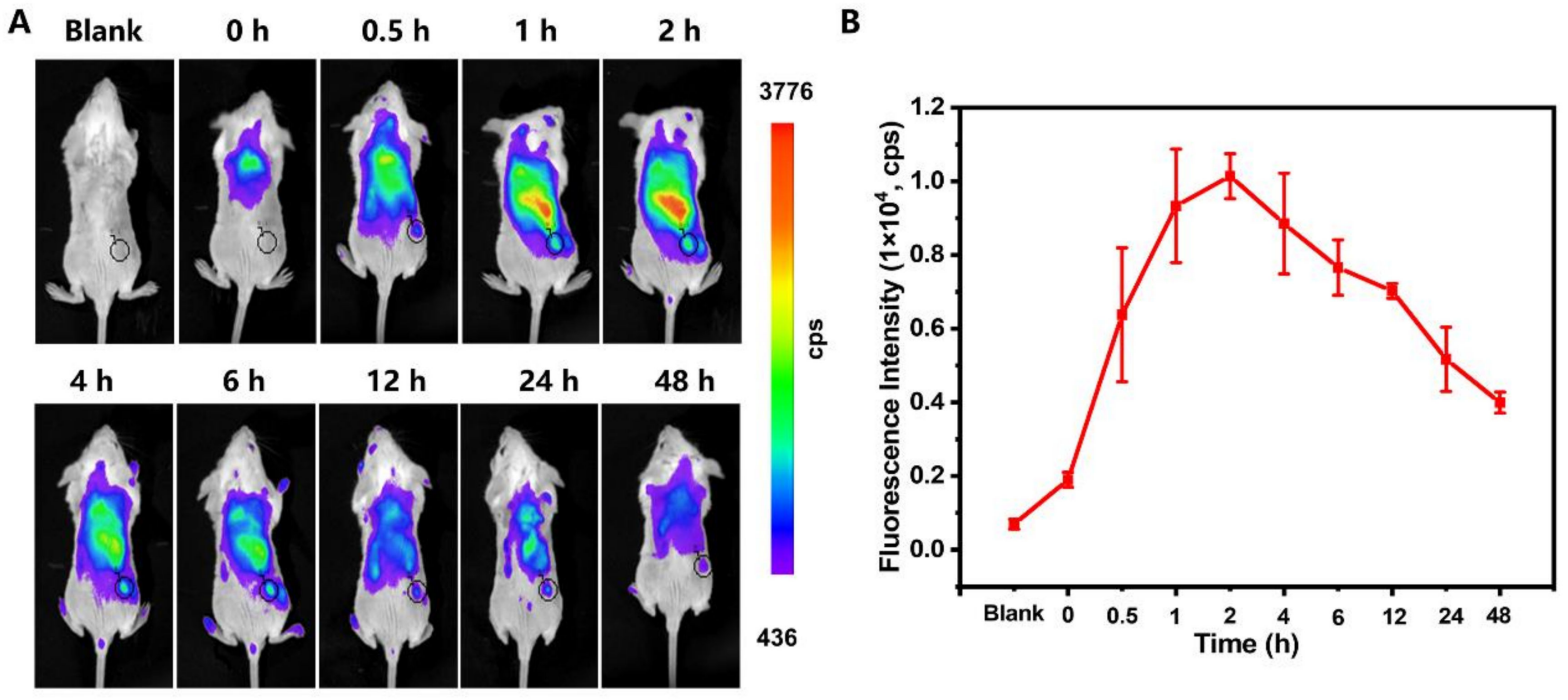
(**A**) In vivo fluorescence imaging of the 4T1 tumor-bearing mice at 0, 0.5, 1, 2, 4, 6, 12, 24, and 48 h after intravenous injection of CLN (2.5 μmol/kg). The black cycles indicate the tumor sites. (**B**) Fluorescence intensities of the tumor sites vs. times (*n* = 3).

Subsequently, the in vivo biodistribution of CLN, following tail-vein injection, was performed on the BALB/c mice bearing 4T1 tumors by an in vivo imaging system. CLN could be effectively accumulated in the tumor site to turn on the fluorescence, exhibiting the strongest fluorescent signal at 2–6 h (Figure 6A). The statistical results of the fluorescence intensities indicated that the optimal temporal window was 2 h post-injection for PDT (Figure 6B). At 24 h post-administration, the ex vivo tissue imaging was performed on a small animal imaging system (Figure S14). The fluorescence signals were mainly observed at liver, spleen, and kidney, indicating that CLN was mainly metabolized by the kidney, liver, and spleen. In addition, fluorescent signals were still present in the tumor area at 24 h, indicating that CLN could accumulate at the tumor site for a longer period. Thus, CLN could be a promising NIR imaging-guided PDT agent for tumor treatment applications.

**Figure 7:**
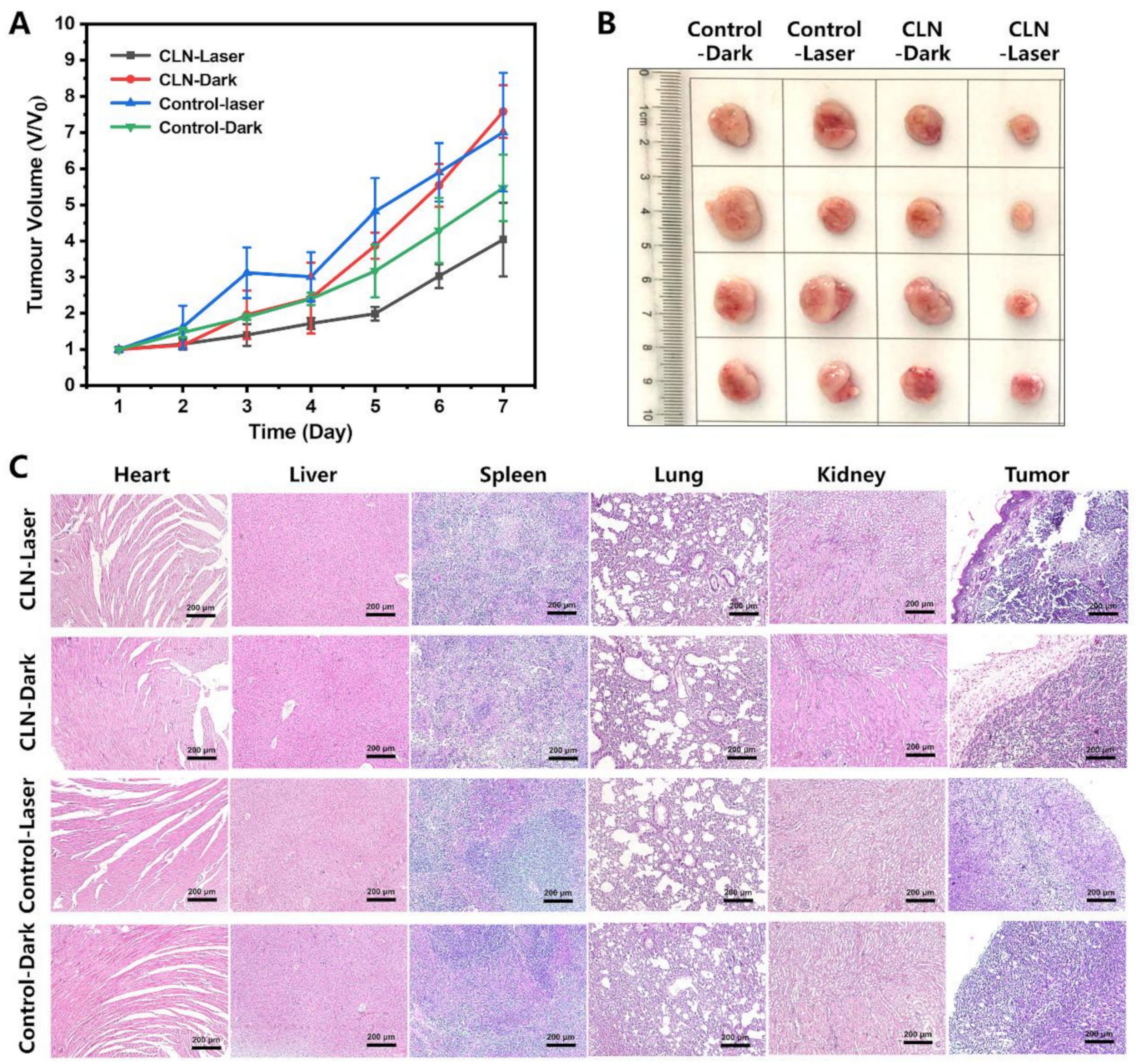
(**A**) Changes in the tumor volumes (*V*_t_/*V*_0_) vs. time in the four groups during the seven days of treatment (*n* = 5; laser, 660 nm; 0.2 W/cm^2^; time, 15 min). (**B**) Photographs of the excised tumors in the four treatment groups. (**C**) H&E analyses of the major organs and tumor tissues for the four groups (Scale bar = 200 µm).

Motivated by the accumulation of CLN, as well as the turned-on fluorescent signal in the tumor regions, we studied its PDT efficiency in vivo. The 4T1 tumor-bearing mice were tail-vein injected with CLN (2.5 μmol/kg) and saline as controls. In the in vivo study, the 4T1 tumor-bearing BALB/c mice were randomly divided into four groups comprising five mice each, and the groups were denoted as “CLN-Laser”, “CLN-Dark”, “Control-Laser”, and “Control-Dark”. Based on NIR imaging results, the optimal phototherapeutic windows were determined after 2 h of injection. Subsequently, all the mice in the “CLN-Laser” and “Control-Laser” groups were exposed to a 660 nm laser (0.2 W/cm^2^) every day after injection for 15 min at day 1, 3, and 5. The in vivo antitumor effects of CLN were investigated by monitoring the changes in the tumor volume for seven days. Figure 7A shows the negligible inhibitions of the tumor growths in the “CLN-Dark”, “Control-Laser”, and “Control-Dark” groups, indicating that irradiation alone did not exert any antitumor effect. Surprisingly, the “CLN-Laser” group revealed the most effective antitumor activity after the treatment, displaying slow tumor growth on day 7. The photographs of the tumor further confirmed the optimal antitumor activity of the “CLN-Laser” group, thereby availing visual evidence (Figure 7B). Additionally, CLN exhibited low in vivo toxicity in the treatments, as further supported by the serum biochemical indices (Figure S13), as well as the hematoxylin and eosin (H&E) staining of the major organs, including the heart, liver, spleen, lung, and kidney of each mouse from all the groups, indicating the absence of significant lesions in the normal organs (Figure 7C). On the seventh day, all four groups of mice were sacrificed to collect their tumor tissues; they were sliced and stained with H&E. The images of the histological sections demonstrated that the “CLN-Laser” group presented the most effective antitumor effect among the different therapeutic agents, effectively inducing tumor necrosis and apoptosis, as well as inhibiting the proliferation of the cancer cells (Figure 7C).

## 3. Materials and Methods

### 3.1. Synthesis

#### 3.1.1. General Information

All reagents were obtained from Energy Chemical Co. Ltd. (Weinan, China) and used as received. Solvents were of analytical grade. Flash column chromatography was performed with silica gel (200–300 mesh) and dichloromethane/methanol was used as eluent. All NMR experiments were measured by using an Agilent 400 DD2 spectrometer (Agilent, Santa Clara, CA, USA) at room temperature. The HRMS were obtained with Electrospray-quadrupole time-of-flight mass spectrometer (MicrOTOF-Q II 10410, Bruker, NASDAQ, Billerica, MA, USA).

#### 3.1.2. Synthesis of Compound **1**

Compound **1** was prepared following a modified procedure of the previous literature [47]. The phosphorous oxychloride (5.0 mL, 53.6 mmol) was added dropwise to the mixed solution of DMF (6.0 mL, 77.6 mmol) and DCM (7 mL) in an ice bath, and stirring was continued in the ice bath for 0.5 h. Then, 4-(4-hydroxyphenyl)cyclohexan-1-one (1.0 g, 5.2 mmol) was added, and the resulting mixture was vigorously stirred and heated to 50 °C for 5 h, cooled to room temperature, and then poured into ice-cooled water 200 mL. The mixture was kept at 4 °C refrigerator overnight and then filtered and freeze dried to obtain compound **1** (0.63 g, 45%).

#### 3.1.3. Synthesis of Compound **2**

The 2,3,3-trimethyl-3H-indole (3.0 g, 19.5 mmol) and *p*-nitrobenzyl bromide (5.4 g, 25.0 mmol) were added to the toluene (30 mL) and refluxed in an oil bath at 90 °C for 12 h; a large amount of purple-red solid was formed, which was cooled to room temperature, filtered under reduced pressure, washed with toluene (5 mL × 3), and dried to obtain 3.9 g of purple-red powder with yield of 70%. The NMR spectra are shown in Supplementary Materials (Figures S1 and S2). ^1^H NMR (400 MHz, DMSO-*d*_6_) *δ* (ppm) 8.20 (d, *J* = 8.5 Hz, 2H, Ar-H), 7.47 (d, *J* = 8.4 Hz, 2H, Ar-H), 7.23 (d, *J* = 7.2 Hz, 1H, Ar-H), 7.06 (t, *J* = 7.6 Hz, 1H, Ar-H), 6.76 (t, *J* = 7.4 Hz, 1H, Ar-H), 6.67 (d, *J* = 7.8 Hz, 1H, Ar-H), 4.94 (s, 2H, -CH_2_-), 3.89 (s, 3H, -CH_3_), 1.34 (s, 6H, -CH_3_); ^13^C NMR (101 MHz, DMSO-*d*_6_) *δ* (ppm) 160.74, 146.87, 146.35, 145.59, 137.16, 128.11, 124.09, 122.51, 119.25, 105.86, 75.58, 44.74, 44.15, 30.21.

#### 3.1.4. Synthesis of Compound **3**

The compound **1** (0.30 g, 1.13 mmol), compound **2** (0.77 g, 2.60 mmol), and anhydrous sodium acetate (0.09 g, 1.13 mmol) were added to the anhydrous ethanol 20 mL, and the mixture was stirred at 80 °C for 4 h, the solvent was evaporated under reduced pressure, the product was purified by using silica gel column chromatography (DCM: MeOH = 50:1) to give green solid 0.38 g, yield 41%. The NMR spectra and HRMS are shown in Supplementary Materials (Figures S3–S5). ^1^H NMR (400 MHz, DMSO-*d*_6_) *δ* (ppm) 9.35 (s, 1H, Ar-OH), 8.41 (d, *J* = 13.9 Hz, 2H, Ar-H), 8.26 (d, *J* = 8.1 Hz, 4H, Ar-H), 7.76 (d, *J* = 6.9 Hz, 2H, Ar-H), 7.55 (d, *J* = 7.8 Hz, 4H, Ar-H), 7.47–7.32 (m, 6H, Ar-H), 7.18 (d, *J* = 7.5 Hz, 2H, Ar-H), 6.78 (d, *J* = 7.5 Hz, 2H, -CH-), 6.49 (d, *J* = 13.9 Hz, 2H, -CH-), 5.80 (d, *J* = 23.9 Hz, 4H, -CH_2_-), 2.98 (d, *J* = 14.6 Hz, 4H, -CH_2_-), 2.87 (t, *J* = 11.1 Hz, 1H, -CH-), 1.83 (s, 12H, -CH_3_); ^13^C NMR (101 MHz, CDCl_3_) *δ* (ppm) 175.03, 151.74, 149.10, 146.18, 143.75, 143.49, 142.44, 136.40, 130.13, 129.17, 129.00, 128.80, 127.02, 125.30, 123.87, 116.38, 112.30, 103.42, 50.91, 48.06, 39.05, 34.89, 28.47; HRMS(ESI-MS) C_50_H_46_ClN_4_O_5_^+^, calculated for [M]^+^ 817.3151, found 817.3169.

#### 3.1.5. Synthesis of CLN

The compound **3** (0.1 g, 0.12 mmol) and N-(2-aminoethylmorpholine) (0.03 g, 0.23 mmol, 30 μL) were added to acetonitrile (10 mL), and the mixture were refluxed in an oil bath at 60 °C for 4 h; the solvent was evaporated under reduced pressure, the product was purified by using silica gel column chromatography (MeOH:DCM = 1:20) to give blue solid 60 mg, yield 54%. The NMR spectra and high-resolution mass spectrometry are shown in Supplementary Materials (Figures S6–S8). ^1^H NMR (400 MHz, DMSO-*d*_6_) *δ* (ppm) 9.24 (s, 1H, -OH), 8.70 (s, 1H, -NH), 8.16 (d, *J* = 6.0 Hz, 4H, Ar-H), 7.48 (dd, *J* = 23.3, 5.8 Hz, 8H, Ar-H), 7.32–7.21 (m, 2H, Ar-H), 7.20–7.01 (m, 4H, Ar-H), 6.91 (d, *J* = 5.1 Hz, 2H, Ar-H), 6.68 (d, *J* = 6.9 Hz, 2H, -CH-), 5.65 (d, *J* = 11.7 Hz, 2H, -CH-), 5.35 (s, 4H, -CH_2_-), 3.82 (s, 1H, -CH-), 3.61 (s, 4H), 2.63 (d, *J* = 17.2 Hz, 4H, -CH_2_-), 2.43 (s, 4H, -CH_2_-), 2.27–2.17 (m, 2H, -CH_2_-), 1.65 (d, *J* = 19.9 Hz, 12H, -CH_3_), 1.24 (s, 2H-CH_2_-); ^13^C NMR (101 MHz, DMSO-*d*_6_) δ (ppm) 166.52, 156.35, 147.20, 144.48, 143.48, 139.84, 137.65, 135.22, 128.67, 128.22, 124.29, 122.99, 122.75, 115.38, 109.28, 95.49, 66.47, 53.50, 47.46, 45.48, 38.07, 33.23, 28.94; HRMS(ESI-MS) C_56_H_59_N_6_O_6_^+^, calculated for [M]^+^ 911.4491, found 911.4421.

### 3.2. Photochemistry

#### 3.2.1. General Information

Na_2_S_2_O_4_ was purchased from Energy Chemical Co. Ltd. (Shanghai, China). SOSG was purchased from Dalian Meilun Biotechnology Co. Ltd. (Dalian, China). NTR was obtained from Sigma-Aldrich Co. Ltd. (Shanghai, China). NADPH was purchased from J&K Scientific, Co. Ltd. (Beijing, China). All UV-Vis spectra were recorded on a TU-1901 spectrophotometer (PuXi Science and Technology Development Co., Ltd., Beijing, China). The fluorescence analyses were performed on a Carry Eclipse fluorescence spectrophotometer (Agilent, Santa Clara, CA, USA). A Laser (660 nm) was obtained from Changchun Femtosecond Technology Co., Ltd., (Changchun, China).

#### 3.2.2. Absorption and Fluorescence Spectroscopy

CLN was dissolved in methanol at a concentration of 1 mM as the stock solution and stored at -20 °C for further use. Na_2_S_2_O_4_ was freshly prepared in 5 mM solution with deionized water and ready to use. The absorption and fluorescence spectroscopy of CLN were performed in PBS (10 mM, pH = 7.4) at a concentration of 10 μM in the absence or presence of Na_2_S_2_O_4_ (0–0.4 mM) at 37 °C. PBS was used for all experiments at 10 mM, pH = 7.4.

#### 3.2.3. Response Mechanism Elucidation

Na_2_S_2_O_4_ was dissolved in D_2_O to a concentration of 1 mM, and 5 mg of CLN was dissolved in DMSO-*d*_6_ and its ^1^H NMR spectrum was tested, then an appropriate amount of sodium hydrosulfite solution was added and then its ^1^H NMR spectrum was tested at 1, 30, and 60 min. Subsequently, this solution was then subjected to HRMS. The spectral data are presented in Supplementary Materials (Figures S9 and S10).

#### 3.2.4. Evaluation of Singlet Oxygen Quantum Yield (Φ_Δ_)

In order to evaluate the photophysical properties of CLN after response, its reduction products (CLNH) were firstly prepared. Briefly, CLNH was separated by using a flash column after reacting with a 1:1 (*V*:*V*) mixture of methanolic solution of CLN (10 mg) and aqueous solution of Na_2_S_2_O_4_ (43.5 mg) for 5 min.

The singlet oxygen quantum yield of CLNH was carried out by surveying the fluorescence intensity of SOSG at 525 nm under laser irradiation for various time. Firstly, the lyophilized powder of SOSG was prepared in methanol to 5 mM stock solution for use according to the instruction and diluted to 100 μM with PBS, further prepared in a 3 mL cuvette to 2 μM working solution, mixed with 10 μM of CLNH in PBS, which was exposed to 660 nm (200 mW/cm^2^) laser lamp irradiation for different time, and the fluorescence spectra were collected by fluorescence spectrophotometer with excitation wavelength of 504 nm. Methylene Blue (MB) was used as the reference. At last, the singlet oxygen quantum yield was calculated with the following Equation (1):

*Φ*_Δ_ = *Φ*_MB_ × (*k*_x_ × *F*_MB_)/(*k*_MB_ × *F*_x_) (1)

*Φ*_Δ_ represents the singlet oxygen quantum yield of the tested photosensitizer; *Φ*_MB_ represents the singlet oxygen quantum yield of MB and the value is 0.39 in water [45]; *x* represents the tested photosensitizer CLNH; *k* represents the slope of the increase in the fluorescence intensity at 525 nm of SOSG with the addition of irradiation time; *F* is the correction factor which is calculated by the following Equation (2):

*F* = 1−10^−OD^ (2)

OD represents the absorbance of the mixture at 660 nm.

#### 3.2.5. Determination of Fluorescence Quantum Yield (*Φ*_f_)

The fluorescence quantum yields of CLN and its reduction product CLNH in PBS were determined using cresyl violet (CV) as the reference. Using 570 nm as the excitation wavelength, the absorbance of CLN and its solution after reaction with Na_2_S_2_O_4_ was adjusted to be less than 0.05 at 570 nm. The fluorescence spectra of CLN, CLNH, and CV were tested under the same conditions and the integrated areas were calculated. The fluorescence quantum yields of CLN and CLNH were calculated by the following Equation (3):

*Φ*_f_ = *Φ*_CV_ × (*I*_x_ (1−10^−*A*cv^))/(*I*_CV_ (1−10^−*A*x^)) (3)

*Φ*_f_ represents the fluorescence quantum yield of the tested photosensitizer; *Φ*_CV_ represents the fluorescence quantum yield of CV and the value is 0.43 in water [48]; *I*_x_ and *I*_CV_ represent integrated areas of the tested PS and CV, respectively; *A*_x_ and *A*_CV_ represent absorbance of the tested PS and CV at 570 nm, respectively.

#### 3.2.6. NTR Recognition and Selectivity

An amount of 1 mg of NTR was dissolved in 10 mL of sterile PBS, divided into 20 lyophilization tubes, and stored in a −20 °C refrigerator until use. NADPH is prepared in deionized water as a 15 mM solution, ready to use. In a 5 mL tube, PBS buffer containing 10% EtOH and 1 mM CLN (30 μL) were mixed, and then 15 mM NADPH (100 μL) was added to obtain a final concentration of 500 µM. An appropriate volume of NTR was added to the sample solution. The final solution volume was adjusted to 3 mL with PBS buffer containing 10% EtOH. After rapid mixing of the solution, it was incubated at 37 °C for 30 min, and transferred to a 10 mm × 10 mm quartz cell. Fluorescence spectra were recorded with *λ*_ex_ = 648 nm.

The selectivity of CLN toward NTR was also tested, including NaCl (10 mM), MgCl_2_ (10 mM), ZnSO_4_ (10 mM), ClO^−^ (10 mM), H_2_O_2_ (10 mM), GSH (1 mM), Vc (1 mM), Cys (1 mM), Gly (1 mM), NADPH (500 μM), and NTR (5 μg/mL, in the presence of 500 μM NADPH). All components were prepared in deionized water and the tests were performed in PBS buffer at 37 °C in the presence of CLN (10 μM) with a 30 min incubation.

### 3.3. Biology

#### 3.3.1. General Information

Fetal bovine serum (FBS) was purchased from AusGeneX (Molendinar, Australia, CAT NO. FBS500-S); DMEM basic (1X) was from Gibco Life Technologies (Shanghai, China); penicillin/streptomycin solution (100 U/mL and 100 mg/mL, respectively), 2′, 7′-dichlorodihydrofluorescein diacetate (DCFH-DA), and Cellular grade DMSO were from Beijing Solarbio Science & Technology Co., Ltd. (Beijing, China); 3-(4,5-dimethyl-1,3-thiazol-2-yl)-2,5-diphenyl-2H-tetrazol-3-ium bromide(MTT) was obtained from Biosharp Life Technology Co., Ltd. (Hefei, China); Hoechest 33342, Mito-Tracker Green, and Lyso-Tracker Green were from Beyotime Biotechnology Co., Ltd. (Shanghai, China). All cells were cultured in a CO_2_ incubator (Wiggens, WCI-180, Beijing, China). All the in vitro fluorescence images experiments were obtained by using an Olympus IX73 + DP73 inverted microscope and laser confocal microscope (Carl Zeiss LSM 900, Jena, Germany). Hypoxic condition was created by coverslips (Citoglas, Hong Kong, China) and a hypoxia chamber (Stemcell Technologies, Cat 27310, Kent, WA, USA). Cytotoxicity assay was performed using an LED light (F&V, Z96kit, Fuijan, China) as irradiation source.

#### 3.3.2. Cell Culture

The 4T1 cells and HeLa cells were cultured in DMEM media with 10% FBS and 1% penicillin/streptomycin solution. Cells were grown in a saturated humidity and 5% CO_2_ atmosphere at 37 °C in a cell incubator.

#### 3.3.3. Fluorescence Imaging in Hypoxia Cells

A cellular hypoxic environment was created by passing the gas mixture (5% CO_2_ + 95% N_2_) through the hypoxia chamber. The 4T1 and HeLa cells were first incubated normally (20% O_2_) at a density of 5 × 10^4^ for 24 h, respectively. Cells were treated with CLN (5 μM) for 3 h and then washed 2–3 times with PBS and fresh medium was added. Cells in the hypoxic group were treated with 0.5 mM NTR inhibitor (dicoumarin) for 1 h and then incubated in the hypoxia chamber with the gas mixture for 8 h at 37 °C in a cell incubator. The normoxic group was incubated normally (20% O_2_) for 8 h and imaged by inverted fluorescence microscopy (Olympus IX73, Tokyo, Japan). Fluorescence intensity analysis was performed by Image J software.

Fluorescence intensity was quantified as follows: 6 cell areas and 3 backgrounds were randomly circled in the image using Image J software, the integrated density of the circled cells and the mean gray value of the background area by Image J software were calculated, and the corrected integrated density of each circled cell was found by the following Equation (4):

IntDen_(corrected)_ = IntDen_(cell)_ − Area_(cell)_ × Mean_(BG)_ (4)

IntDen_(corrected)_ represents the integrated density of each circled cell deducting the mean gray value of the background; IntDen_(cell)_ indicates the total fluorescence intensity of the region; Area_(cell)_ indicates the area of the cell; and Mean_(BG)_ indicates the mean gray value of the background region (the average of mean of the three background regions).

#### 3.3.4. Subcellular Colocalization Fluorescence Imaging of Cells

The HeLa cells were seeded in a glass bottom dish and incubated for 24 h under normoxia condition (20% O_2_) at a density of 5 × 10^4^. Then, the cells were incubated for another 8 h under hypoxia condition with coverslips. Next, cells were treated with CLN (5 μM) for 3 h and then washed 2–3 times with PBS and fresh medium with Hoechest 33,342 (1×) and Lyso-Tracker Green (or Mito-Tracker Green, respectively) (300 nM) and incubated for 30 min. Last, these cells were imaged by laser confocal microscope (Carl Zeiss LSM 900). The blue channel was excited at 405 nm, the green channel was excited at 488 nm, and the red channel was excited at 640 nm. Colocalization analysis was performed by Image J software.

#### 3.3.5. Detection of Cellular ROS within DCFH-DA

The 4T1 and HeLa cells were seeded on a culture dish at a density of 5 × 10^4^ for 24 h. CLN (5 μM) was added and incubated for 3 h. Then, the cells were washed with PBS 2–3 times and then treated with DCFH-DA (5 μM) for 30 min and imaged in the presence or absence of a laser (660 nm, 200 mW/cm^2^) irradiated for 10 min under an inverted fluorescence microscopy (Olympus IX73). In addition, NAC (N-Acetyl-L-cysteine) was used as a reactive oxygen scavenger, and after incubation with CLN, the cells were treated with NAC (5 mM) for 1 h, then DCFH-DA was added, and imaging was performed after light exposure. They were washed 2–3 times with PBS after each change in incubation medium, and the rest of the operation was unchanged.

#### 3.3.6. Cytotoxicity Assay

Cytotoxicity assays in the presence or absence of light were performed by reducing MTT. Firstly, 4T1 cells were grown in a 96-well plate at a density of 5 × 10^4^, and incubated under normoxia for 24 h, and then incubated in a hypoxia chamber for another 8 h, treated with different concentrations of CLN (0–12 µM, containing 1% DMSO) for 4 h, and the light group was irradiated by a 96-well LED light (220.7 mW/cm^2^) for 10 min and continued to be incubated until 24 h under hypoxia. As for the dark group, the cells were kept out of light with the other treatments unchanged. Lastly, these cells were incubated with 100 µL MTT (5 mg/mL in DMEM) solution for 4 h at 37 °C in cell incubator, and then the medium was removed carefully, 100 µL of DMSO was added to each well, and the absorbance was tested at 490 nm on a microplate reader. The cell viability was calculated according to the following Equation (5):

Cell viability% = (OD_sample_ − OD_blank_)/(OD_control_ − OD_blank_) × 100% (5)

### 3.4. Animal Assays

#### 3.4.1. General Information

All procedures were carried out in accordance with the interrelated legislation and ethical regulation guidelines were endorsed by the Model Animal Research Center of Zunyi Medical University. The experimental animals were 18~22 g BALB/c female mice, purchased from Spelford Beijing Biotechnology Co., Ltd. (SPF grade, license number: SCXK (Beijing) 2016-0002). The BALB/c mice were first adaptively housed for 1 week, the dorsal hair was shaved, and then they were inoculated with 100 μL of 4T1 cells at a density of 1 × 10^7^ at the lower right position of their back, normal rearing was continued for 3~5 days, tumor growth observed, and the experiment was performed as the tumor grew to 80 mm^3^. All CLN injections were 500 μM of CLN solution (Tris buffer, 10 mM, pH = 7.4, containing 10% DMSO) administered at a dose of 2.5 μmol/kg. All in vivo imaging experiments were performed on a NightOWL II LB983small animal imaging system (Berthold Technologies Gmbh & Co. KG).

#### 3.4.2. In Vivo Imaging of Mice with Endogenous NTR

Six tumor-bearing BALB/c mice were randomly divided into two groups. One group of tumor-bearing mice was injected intratumorally with CLN at a dose of 2.5 μmol/kg, the other mice were injected intratumorally with 100 µL dicoumarin (500 μM) for 10 min before the injection of CLN (2.5 μmol/kg). The time-dependent fluorescence changes were collected at every 20 min within 120 min after injection with a small animal imaging system. The excitation wavelength was 640 nm, and the emission wavelength was 820 nm.

#### 3.4.3. In Vivo Fluorescence Imaging and Tumor Targeting

Three tumor-bearing BALB/c mice were randomly selected and injected with CLN at a dose of 2.5 μmol/kg in the caudal vein, and imaged with a small animal imaging system at various times. The excitation wavelength was 640 nm, and the emission wavelength was 820 nm.

#### 3.4.4. The Photocytotoxic Efficiency of Tumor-Bearing Mice

Twenty tumor-bearing BALB/c mice were randomly divided into four groups: “Control-laser group”, “Control-dark group”, “CLN-laser group”, and “CLN-dark group”. The control group was injected with 100 μL saline via tail-vein injection, the CLN group was injected with CLN at a dose of 2.5 μmol/kg, and the “CLN-laser group” was irradiated with a 660 nm (0.2 W/cm^2^) laser for 15 min after treated with CLN via tail-vein injection for 2 h. The study period was 7 days, and the CLN was injected once a day at an interval, three times within 7 days, and the laser groups were irradiated for 15 min each day. The mice were weighed, and the tumor volume was measured and recorded daily. The tumor volume was measured by vernier calipers and calculated according to the following Formula (6):

*V* = 1/2 × *a* × *b*^2^ (6)

*a* denotes the longest diameter of the tumor, and *b* represents the vertical direction diameter of the tumor according to *a*.

At the end of the study on day 7, the tumor tissues were taken for size measurement and H&E staining. Blood was collected from each mouse, and serum was collected to measure serum biochemical indices: aspartate aminotransferase (AST), glutamate transferase (ALT), creatinine (CREA), and urea (UREA), and compared with normal mice for biochemical parameters. The reference ranges of biochemical parameters in normal healthy BALB/c female mice were: ALT (38.31 ± 15.07 U/L), AST (151.2 ± 66.6 U/L), CREA (11.27 ± 4.83 μmol/L), UREA (7.49 ± 1.65 mmol/L) [49]. The serum biochemical data are presented in Supplementary Materials (Figure S12).

#### 3.4.5. H&E Staining

The heart, liver, spleen, lung, kidney, and tumor tissues of the four groups of mice were taken and fixed with 4% formaldehyde solution, and then dehydrated and embedded with paraffin to slice the 4 µm sections, which were conducted for hematoxylin and eosin (H&E) under standard methods and sent to a microscope (Olympus CX41).

## 4. Conclusions

In summary, we designed and synthesized a new cyanine-based PS, CLN, which was modified with nitrobenzene and morpholine groups. The maximum absorption wavelength of CLN was located in the red-light region. As expected, almost no fluorescence was detected before the CLN response. However, in the presence of Na_2_S_2_O_4_, a fluorescence signal appeared in the NIR region, and the fluorescence intensity at 758 nm was 71 times higher than before. The mechanism of the generation of fluorescence was confirmed by NMR titration and MS. The MS results demonstrated that the nitro group was reduced into amino to produce a new compound, CLNH. Further, the calculated singlet oxygen quantum yield of CLNH was 1.60%. Similarly, the fluorescence of CLN also exhibited a specific response to NTR in a complicated environment. The fluorescence of CLN could be selectively turned on in hypoxic cells. Cellular fluorescence colocalization experiments revealed that CLN targeted the lysosome effectively. Thus, selective imaging and targeted PDT were concurrently achieved in vitro and in vivo.

Taking an upregulated level of NTR as the tumor biomarker [25], CLN achieves a selective turn-on of fluorescence, thus facilitating precise PDT in potential clinical applications. In terms of available results, perhaps a local application of CLN may be more effective, as CLN is limited by tumor-targeting ability and photodynamic effects under hypoxic conditions. Of course, as a potential PS, the molecular structure and properties of CLN need to be improved if used in the clinic. The following improvement strategies may further increase the potential for clinical application of CLN: (1) enhancement of singlet oxygen quantum yield; (2) development of Type I PDT to reduce the dependence on oxygen concentration; (3) construction of oxygen self-sufficient photosensitizers (e.g., modified perfluorocarbon structures); and (4) modification of tumor-targeting groups to enhance PS concentration in the tumor region. The above improvements may be beneficial for clinical use, and in the future, we expect to develop a PS that can be used in the clinic.

## Data Availability

The data presented in this study are available in the Supplementary Materials.

## Supplementary Materials

The following supporting information can be downloaded at: https://www.mdpi.com/article/10.3390/molecules27113457/s1, Figures S1, S3 and S6: The ^1^H NMR spectra of the compounds 2, 3, and CLN; Figures S2, S4 and S7: The ^13^C NMR spectra of the compounds 2, 3, and CLN; Figures S5, S8 and S10: Mass spectra of the compounds 3, CLN, and CLNH; Figure S9: NMR titration spectra of CLN and sodium dithionite; Figure S11: Absorption and fluorescence spectra of CLN at varied pH; the pKa fitting curve and the fluorescence spectra of CLNH in PBS buffer and PBS buffer/10% EtOH; Figure S12: MTT tests of 4T1 cells; Figure S13: Serum biochemical values; Figure S14: Fluorescence imaging and intensity of major ex vivo organs and tumor; Table S1: The optical data of CLN and CLNH in PBS; Table S2: The summary of photophysical properties of reported similar probes.

## Sample Availability

Samples of the compounds all are available from the authors.
