# Supplementary material for: Novel Lysosome-Targeting Fluorescence Off-On Photosensitizer for Near-Infrared Hypoxia Imaging and Photodynamic Therapy In Vitro and In Vivo
Source: Molecules. 2022 May 27;27(11):3457. doi: 10.3390/molecules27113457 (PMC9182569; doi:10.3390/molecules27113457)
Supplement: Supplementary file 1 [file molecules-27-03457-s001.zip › molecules-1707884-supplementary.pdf]

Molecules

Supplementary Materials

Novel Lysosome-Targeting Fluorescence Off-On Photosensitizer for Near-Infrared Hypoxia Imaging and Photodynamic Therapy *In Vitro* and *In Vivo*

Shangli Ding, Mingyan Yang, Jiajia Lv, Hongyu Li, Gang Wei*, Jie Gao,* and Zeli Yuan*

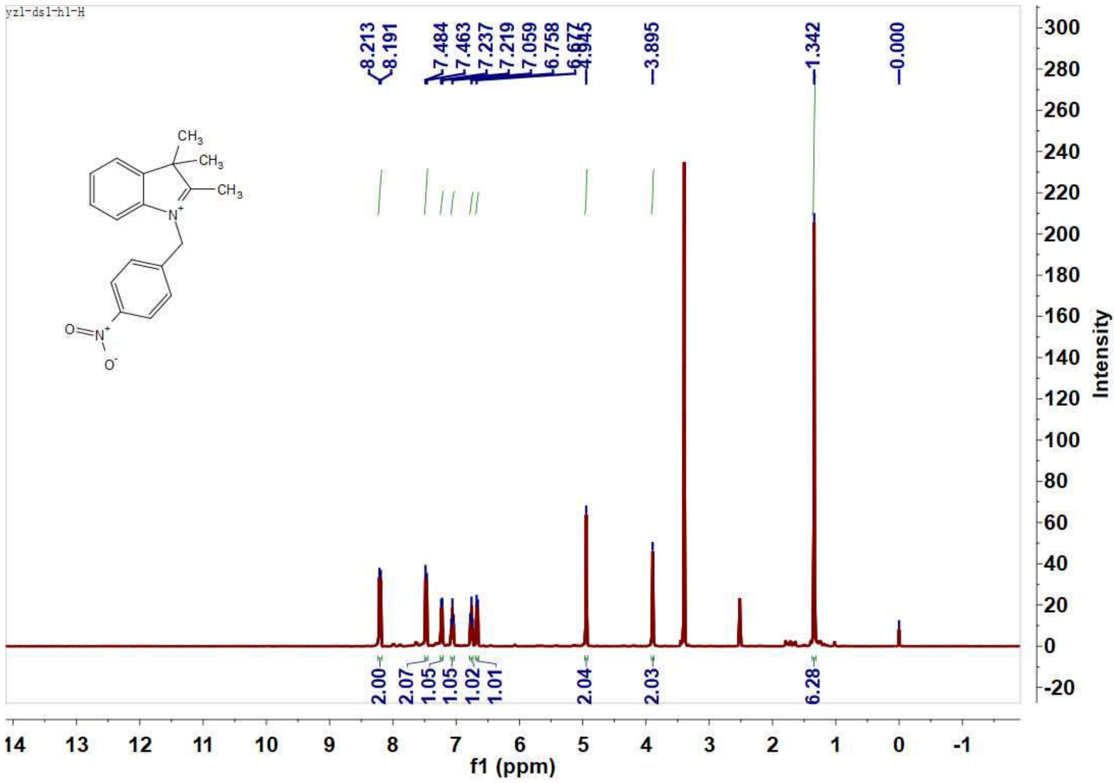

Figure S1 ^{1}H NMR spectrum of compound 2 in DMSO-d_6

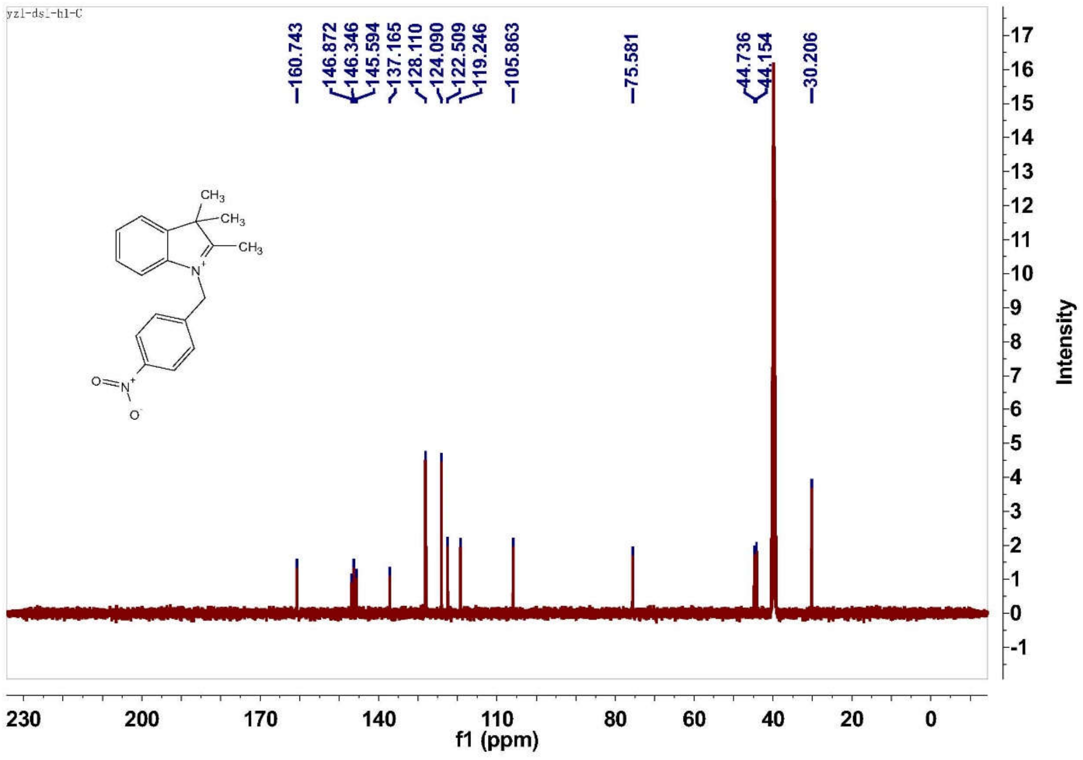

Figure S2 ^{13}C NMR spectrum of compound 2 in DMSO-d_6

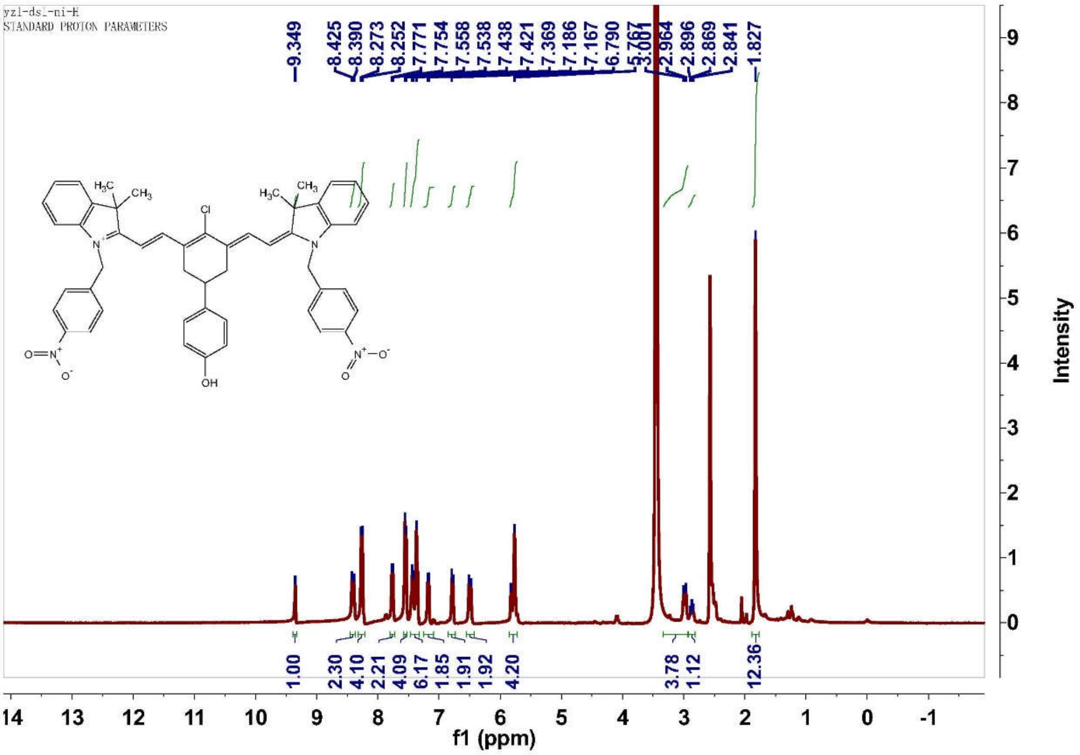

Figure S3 ^{1}H NMR spectrum of compound 3 in DMSO-d_6

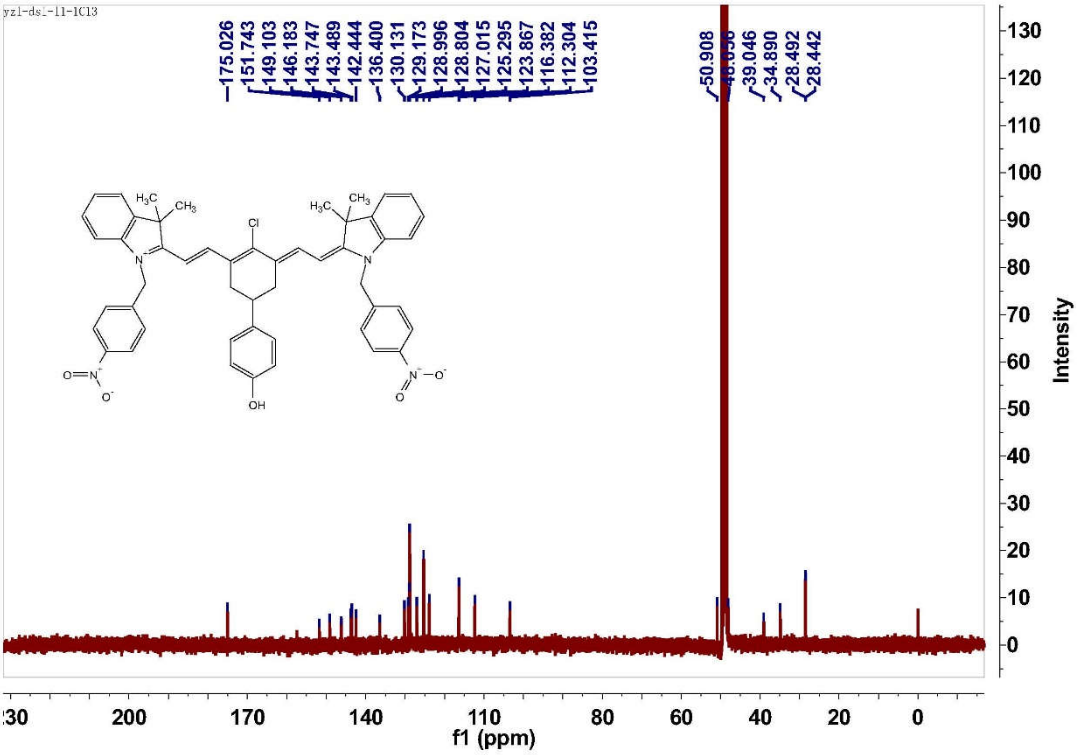

Figure S4 ^{13}C NMR spectrum of compound 3 in DMSO-d_6

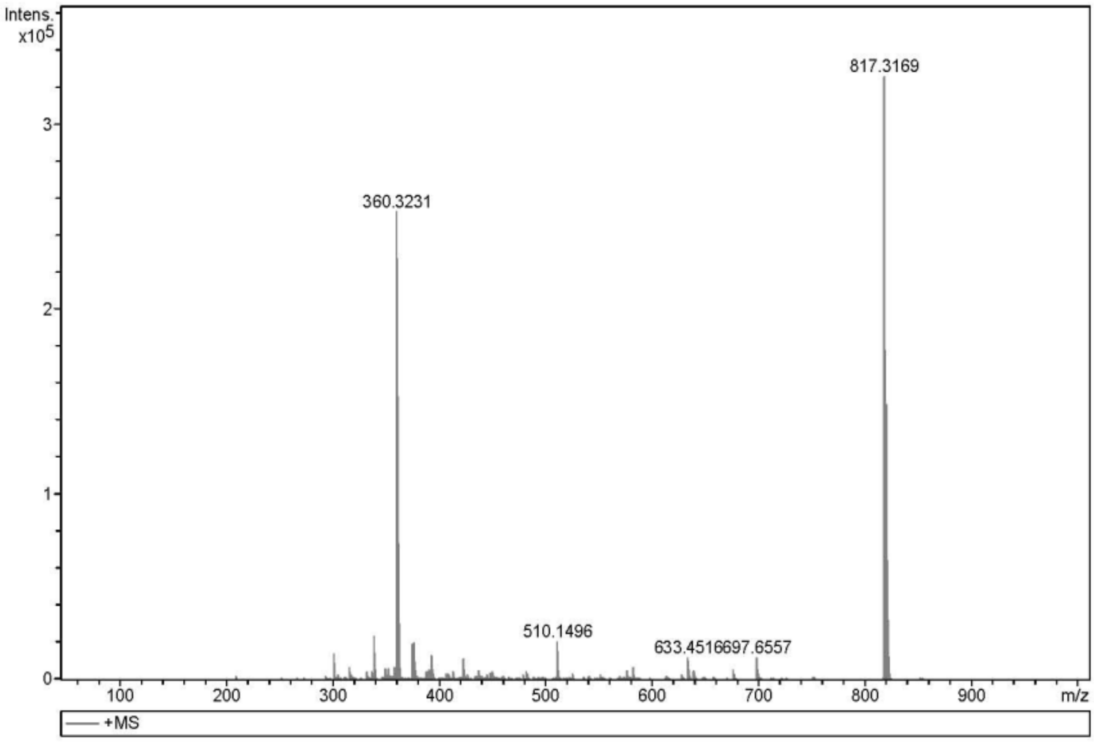

Figure S5 ESI-Mass spectrum of the compound 3

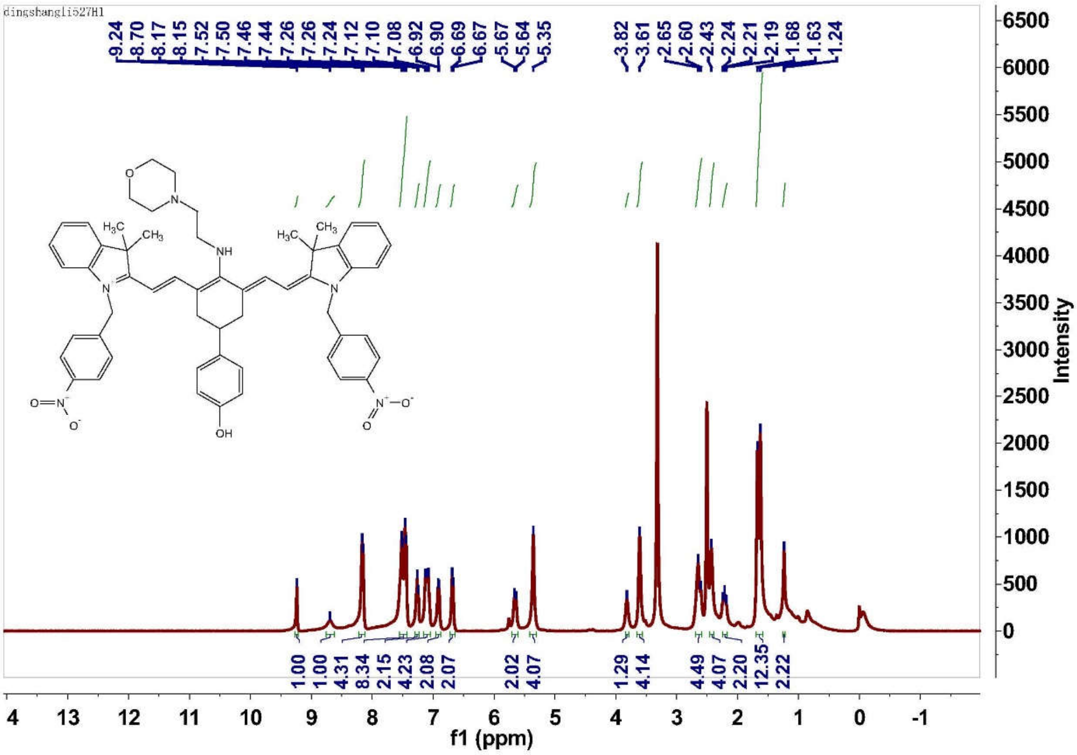

Figure S6 ^{1}H NMR spectrum of compound CLN in DMSO-d_6

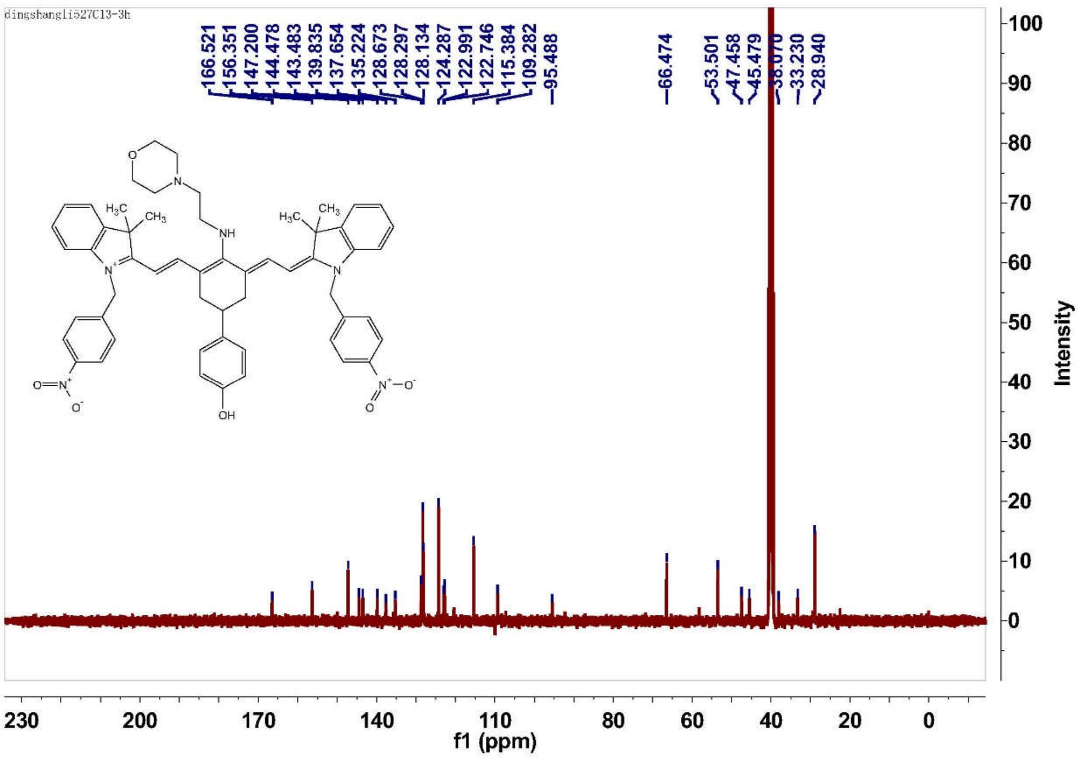

Figure S7 ^{13}C NMR spectrum of compound CLN in DMSO-d_6

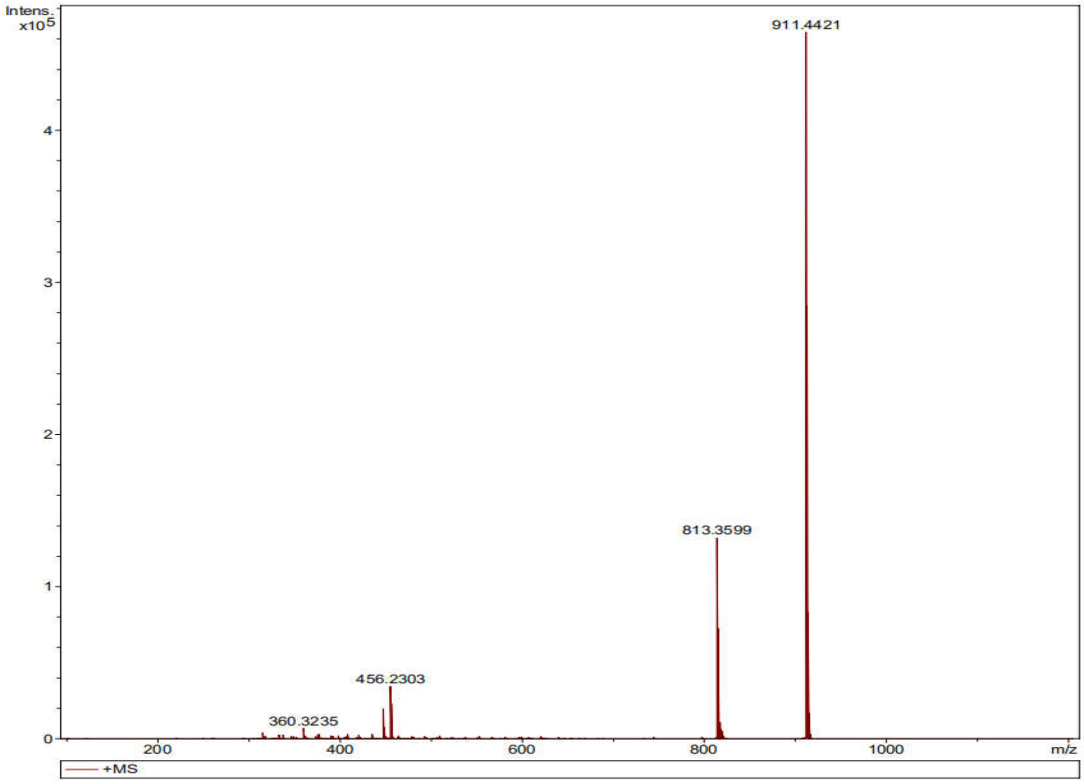

Figure S8 ESI-Mass spectrum of the compound CLN

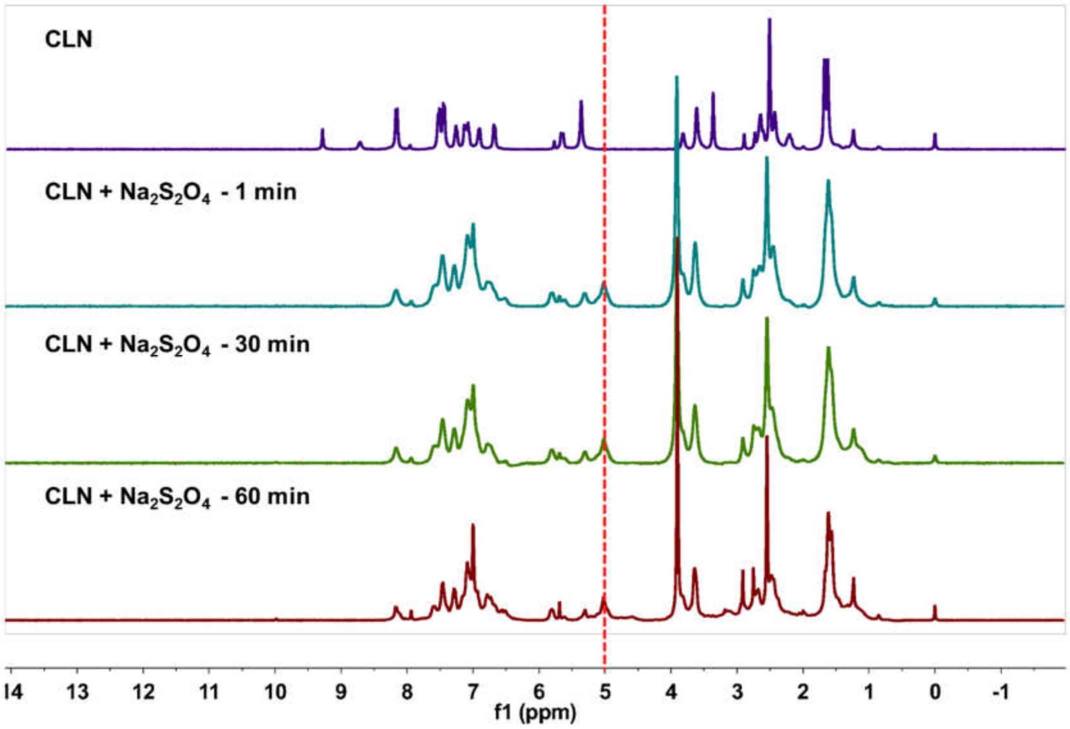

Figure S9 NMR titration spectrum of CLN (5 mg) with $Na_2S_2O_4$ in DMSO-d_6. The red line marked the presence of a new NMR signal at 5. 00 ppm.

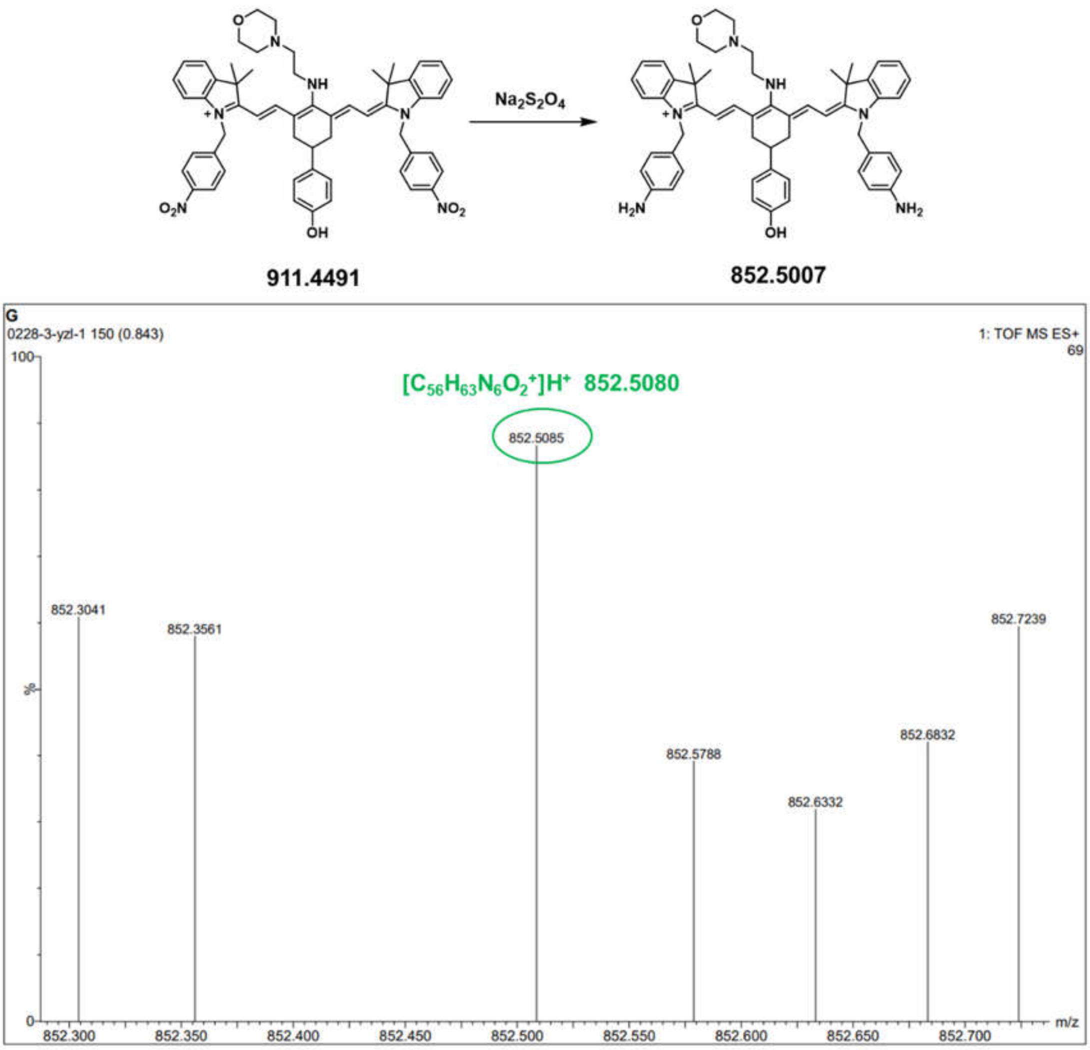

Figure S10 The HRMS spectrum of the compound CLNH.

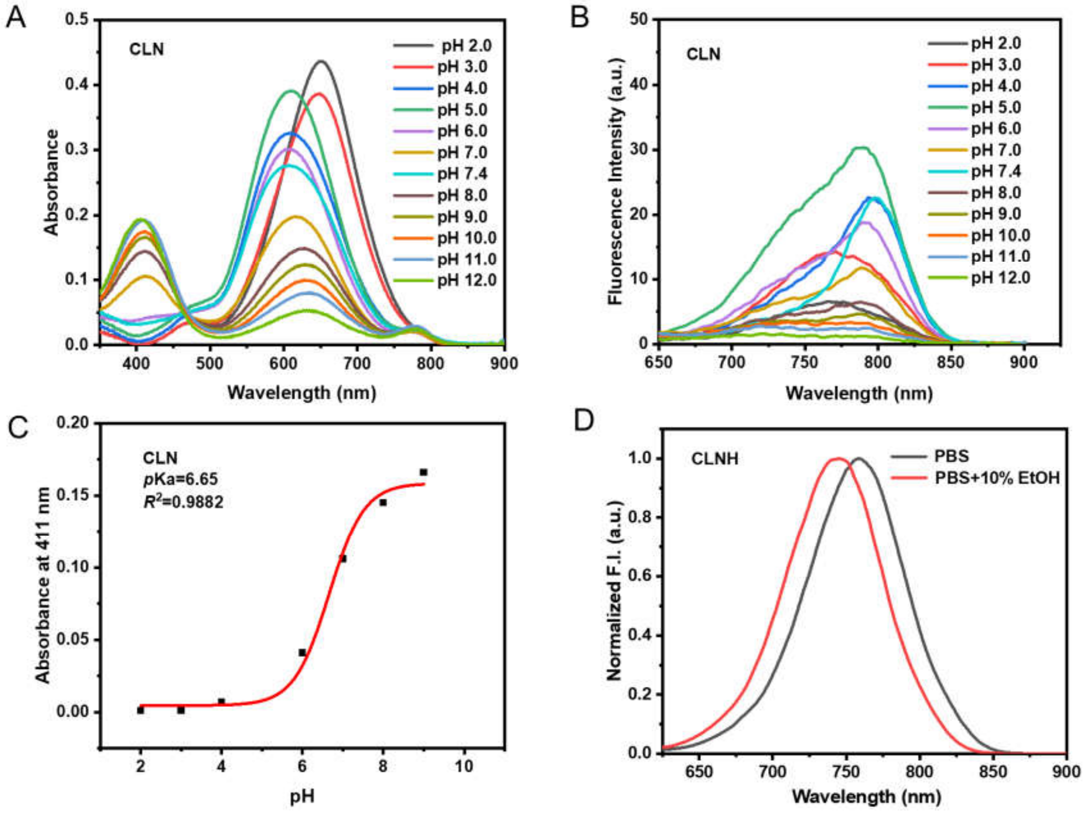

Figure S11 Absorption (A) and fluorescence (B) spectra of CLN (10 μM) at varied pH (2.0-12.0) in 10 mM phosphate buffer. (C) The pKa fitting curve of CLN. (D) The fluorescence spectra of CLNH in PBS buffer and PBS buffer/10% EtOH.

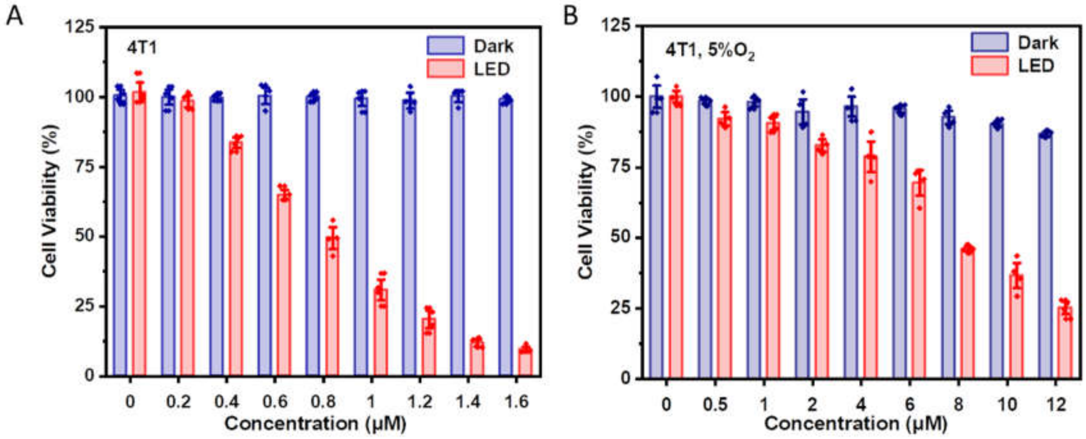

Figure S12 Cytotoxicity of the CLN against 4T1 cells in the absence and presence of LED light irradiation. The cells were treated in a normoxic (A) and hypoxia (B) environment. Cells without any treatment were used as control. Error bars represent the standard derivations of four independent studies.

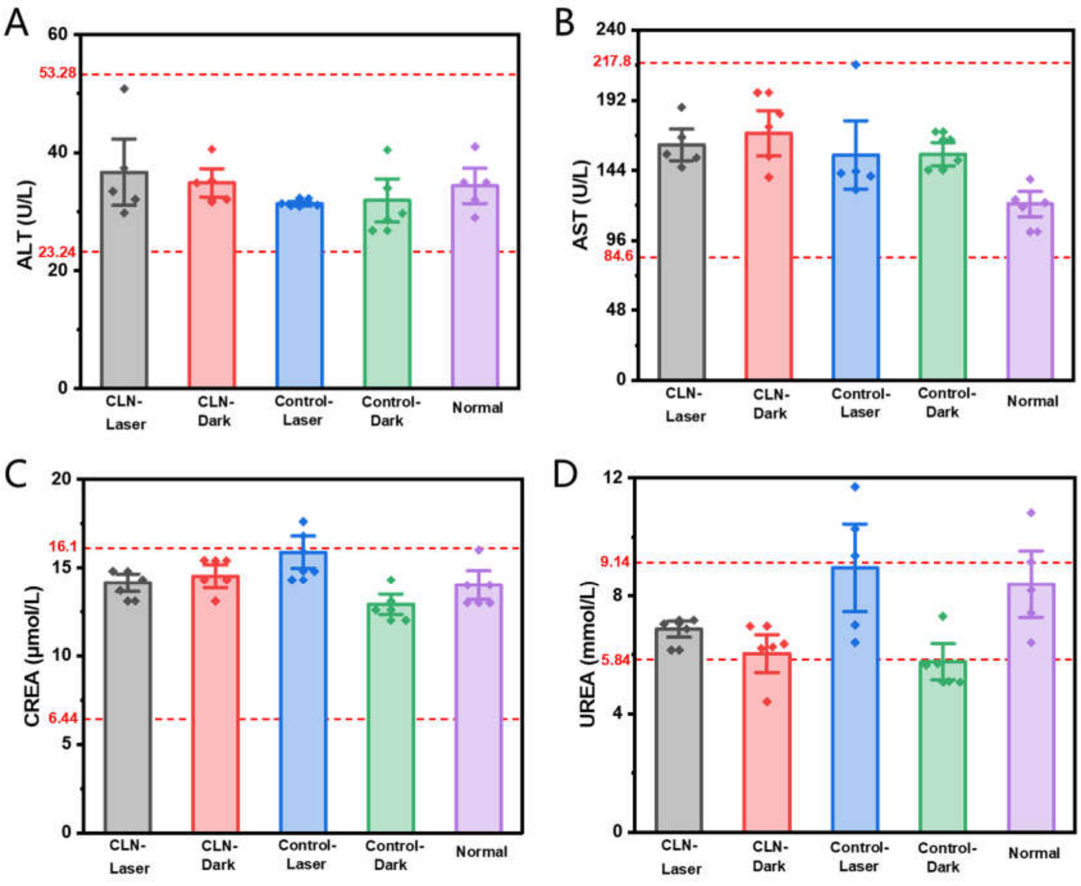

Figure S13 The serum biochemical indices including glutamate transferase (ALT, A), aspartate aminotransferase (AST, B), creatinine (CREA, C) and urea (D) of "CLN-Laser", "CLN-Dark", "Control-Laser" and "Control-Dark" groups. Mice without any treatment were used as control ("Normal" group). The red line marks the normal biochemical index range.

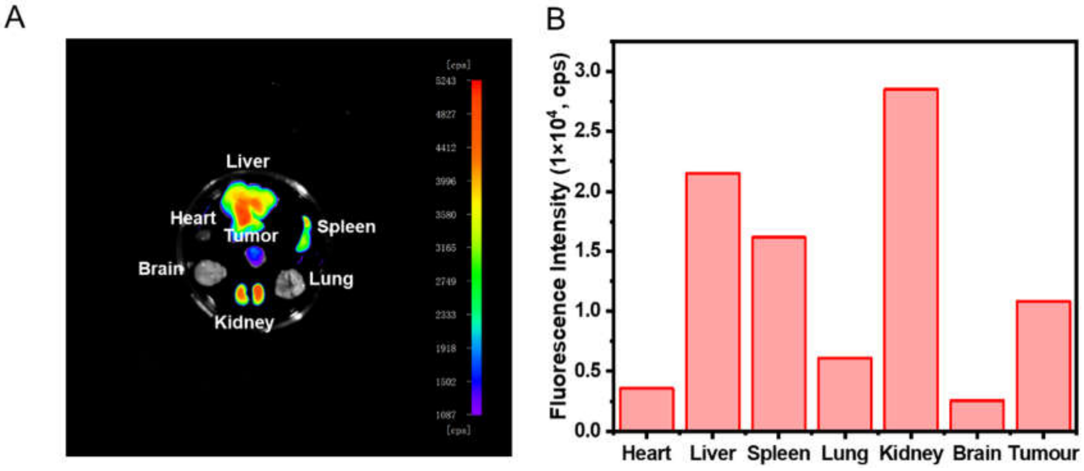

Figure S14 (A) Bio-distribution fluorescence imaging of the 4T1 tumor-bearing BALB/c mice after intravenous injection of CLN (2.5 μmol/kg) for 24 h. (B)

Fluorescence intensity in the heart, liver, spleen, lung, kidney, brain, and tumor of the 4T1 tumor-bearing BALB/c mice after intravenous injection of CLN (2.5 μmol/kg) for 24 h.

Table S1 The optical data of CLN and CLNH in PBS

**PS**	**Solvent**	λ_{abs} **(Max) (nm)**	λ_{em} **(Max) (nm)**	**Stokes shifts (nm)**	ε **(M^{-1} cm^{-1})**	Φ_f **(%)**	Φ_Δ **(%)**
CLN	PBS	611	797	186	20480	0.13	3.90
CLNH	PBS	623	758	135	32860	8.65	1.60

Table S2 The summary of photophysical properties of reported similar probes

NO	Strcture	λabs/λem (nm)[a]	Stocks shift (nm)	Φ_f(%)[b]	Φ_Δ(%)[b]	ε (M^{-1} cm^{-1})[b]	Application	Ref
1	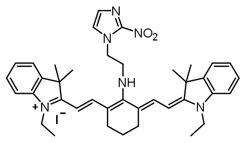	695/750 (PBS)	55	0.03/– (PBS)	–/–	–/–	HepG2 cells	Chem. Commun. 49 (2013) 2554–2556
2	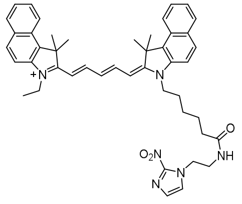	658/699 (Tris buffer)	41	–/–	–/–	–/4600 (Tris buffer)	Bacterial	Chem. Commun. 53 (2017) 11177–11180
3	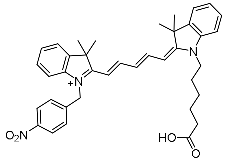	652/677 (Tris buffer)	25	–/–	–/–	–/–	Bacterial	Talanta. 205 (2019) 120133

4	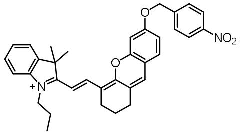	Visible region/–<br>670/705<br>(PBS)	35	–/–	–/–	–/–	Zebrafish	Biosens. Bioelectron. 63 (2015) 112–116
5	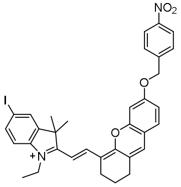	575/–<br>655/710<br>(PBS)	55	–/–	0.72/1.80<br>(DCM)	–/–	PDT, 4T1 cells	Chem. Sci. 10 (2019) 10586–10594
6	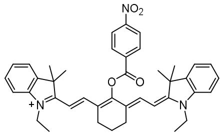	764/–<br>764/782<br>(Tris buffer)	18	< 0.1/1.8<br>(Tris buffer)	–/–	–/–	A549 cells and mouse	J. Am. Chem. Soc. 137 (2015) 6407–6416
7	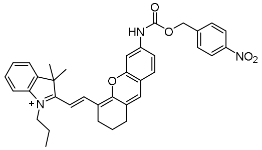	570/–<br>670/706<br>(PBS)	36	–/–	–/–	–/–	Living cells, mouse	Anal. Chem. 88 (2016) 5610–5614

8	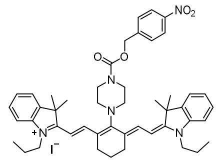	630/785 (PBS)	155	–/–	–/–	–/–	Living cells, mouse	Biosens. Bioelectron. 119 (2018) 141–148
9	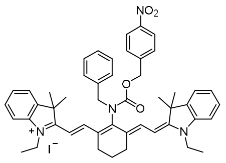	782/805<br>645/747 (PBS)	23<br>102	1.1/3.42 (PBS)	–/–	146000/– (PBS)	A549 cell	Sensors Actuators, B Chem. 222 (2016) 419–424
10	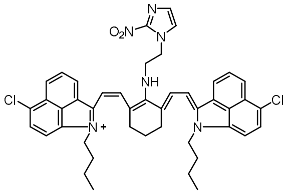	980/1046 (HEPES)	66	1.88×10^{-3}/0.6 (Ethanol)	–/–	–/–	Mice, PA, PTT	Theranostics. 8 (2018) 6025–6034

a: The two sets of $\lambda_{abs}/\lambda_{em}$ are given where the two sets of absorption and emission before and after the reaction of the probe with nitroreductase, respectively, where "–" indicates values not given in the article.

b: In the statistics of Φ_f, Φ_Δ, ε, "x/x" indicates the two test values before and after the reaction of the probe with nitroreductase, where "–" indicates the value not given in the article.
